# Wellbeing of Indigenous Peoples in Canada, Aotearoa (New Zealand) and the United States: A Systematic Review

**DOI:** 10.3390/ijerph18115832

**Published:** 2021-05-28

**Authors:** Alana Gall, Kate Anderson, Kirsten Howard, Abbey Diaz, Alexandra King, Esther Willing, Michele Connolly, Daniel Lindsay, Gail Garvey

**Affiliations:** 1 Wellbeing and Preventable Chronic Disease Division, Menzies School of Health Research, Charles Darwin University, Casuarina, NT 0810, Australia; kate.anderson@menzies.edu.au (K.A.); abbey.diaz@menzies.edu.au (A.D.); Daniel.lindsay@menzies.edu.au (D.L.); gail.garvey@menzies.edu.au (G.G.); 2 School of Public Health, Faculty of Medicine and Health, University of Sydney, Sydney, NSW 2006, Australia; kirsten.howard@sydney.edu.au; 3 Department of Medicine, College of Medicine, University of Saskatchewan, Saskatoon, SK S7N 2Z4, Canada; alexandra.king@usask.ca; 4 Kōhatu–Centre for Hauora Māori, University of Otago, Dunedin 9054, New Zealand; esther.willing@otago.ac.nz; 5 International Group for Indigenous Health Measurement, Columbia, MD 21045, USA; michelebabb@verizon.net

**Keywords:** indigenous health and wellbeing, First Nations, indigenous people/s, wellbeing, well-being, culture, quality of life, QoL

## Abstract

Despite the health improvements afforded to non-Indigenous peoples in Canada, Aotearoa (New Zealand) and the United States, the Indigenous peoples in these countries continue to endure disproportionately high rates of mortality and morbidity. Indigenous peoples’ concepts and understanding of health and wellbeing are holistic; however, due to their diverse social, political, cultural, environmental and economic contexts within and across countries, wellbeing is not experienced uniformly across all Indigenous populations. We aim to identify aspects of wellbeing important to the Indigenous people in Canada, Aotearoa and the United States. We searched CINAHL, Embase, PsycINFO and PubMed databases for papers that included key Indigenous and wellbeing search terms from database inception to April 2020. Papers that included a focus on Indigenous adults residing in Canada, Aotearoa and the United States, and that included empirical qualitative data that described at least one aspect of wellbeing were eligible. Data were analysed using the stages of thematic development recommended by Thomas and Harden for thematic synthesis of qualitative research. Our search resulted in 2669 papers being screened for eligibility. Following full-text screening, 100 papers were deemed eligible for inclusion (Aotearoa (New Zealand) *n* = 16, Canada *n* = 43, United States *n* = 41). Themes varied across countries; however, *identity*, *connection*, *balance* and *self-determination* were common aspects of wellbeing. Having this broader understanding of wellbeing across these cultures can inform decisions made about public health actions and resources.

## 1. Introduction

Canada, Aotearoa (New Zealand) and the United States are all highly developed countries [1], yet the benefits of their development are not shared equally across all members of these societies. Disparities experienced by Indigenous people are underpinned by the unequal distribution of social, political and economic determinants of health and wellbeing [2]. Indigenous peoples’ personal and collective experiences of disempowerment, marginalisation, loss of land, and racism have adversely impacted their health and wellbeing [1,3,4,5]. These factors are exacerbated by institutional systems, including health systems, which generally do not reflect the holistic worldviews or practices of Indigenous peoples [6].

Indigenous populations across these countries live in substantially different social, political, cultural, environmental and economic contexts [3]. Similarly, different terms are used across countries to describe Indigenous peoples such as First Peoples, First Nations, Tribal, Native and Māori. In this paper, we use ‘Indigenous’ to collectively and respectfully describe Indigenous peoples from across Canada, Aotearoa (New Zealand), and the United States [2]. Despite the rich diversity within and between Indigenous peoples in Canada, Aotearoa (New Zealand), and the United States, Indigenous paradigms commonly embrace holistic and collectivist conceptions of health and wellbeing [7]. These paradigms are grounded in Indigenous people’s worldviews, which are formed over one’s lifespan through socialisation and social interactions [6]. Indigenous worldviews are often heavily shaped by the environment, owing to the unique and intricate relationships they hold with the land and sea [6]. This extends further to a relational worldview that encompasses Indigenous people’s spirituality and the importance of their collectivist cultures (communities and tribes) with respectful individualism, whereby the individual thinks not only about themselves when making decisions, but rather, the whole community [6].

In order to make appropriate decisions about the allocation of public health resources to address health and wellbeing inequities, we first need to understand what aspects of health and wellbeing are valued by and relevant to Indigenous peoples. Understandings of health and wellbeing are culturally bound [8,9]. Globally, there is a growing interest in identifying aspects of wellbeing that are valued by Indigenous populations. Existing measures of wellbeing that underpin many health interventions have been developed within Western philosophical constructs that do not include Indigenous conceptions of wellbeing [10]. There is an imperative to understand wellbeing from an Indigenous cultural perspective and for measures to be developed that assess indicators that have a more holistic view of health. In Australia, a recent systematic review by Butler et al. has shed some light on aspects of wellbeing that are important to and valued by Indigenous Australians (see Box 1 for details) [11]. To date, the broad aspects of wellbeing for Indigenous peoples in Canada, Aotearoa (New Zealand) and the United States have not been systematically reported. In this study, we aim to identify aspects of wellbeing important to the Indigenous peoples in Canada, Aotearoa (New Zealand) and the United States, and highlight any similarities and differences across these countries.

Box 1 **Butler et al., 2019. Domains of wellbeing for Indigenous Australians [11].**

(1) Autonomy, empowerment, and recognition; which influences wellbeing through agency and self-determination of the individual and community;

(2) Family and community; including kinships and notions of social and cultural connectedness;

(3) Culture, spirituality and identity; which is interrelated and multidirectional;

(4) Country; which is a holisticconcept encompassing identity, spirituality, culture, peoples, language, law and ceremony;

(5) Basic needs; including food, money, housing and access to services;

(6) Work, roles and responsibilities; both paid employment to meet basic needs and roles within community (including employment with Indigenous led companies);

(7) Education; which presented as a complex relationship where tensions were felt between the need for formal schooling, and desire to pass on and learn cultural knowledge, and;

(8&9) Physical health and Mental health (two separate themes); both encompassed a holistic understanding of health that is complex and culturally-bound.

## 2. Materials and Methods

The current review was led by an Indigenous researcher from Australia (AG), with guidance from senior Indigenous researchers from Australia (GG), Canada (AK), Aotearoa (New Zealand) (EW) and the United States (MC). This review is part of a larger body of work that aims to develop a new instrument to measure wellbeing for Aboriginal and Torres Strait Islander adults, in order to more effectively inform decision making to improve health and wellbeing (the ‘What Matters 2 Adults’ Research Program [12]). The WM2Adults Research Program is guided by an Indigenous Project Advisory Group in Australia.

### 2.1. Eligibility Criteria

Peer-reviewed, indexed, and published papers that included Indigenous adults (18+ years) residing in Canada, Aotearoa (New Zealand) and the United States, and that qualitatively examined at least one aspect of wellbeing for a general (non-disease specific) Indigenous population, were eligible. There were no restrictions based on qualitative methodology or study quality.

We excluded papers reporting an intervention or trial (where the focus was only on the outcomes), in which the findings applied only to a specific disease or condition, those that focused on young people under the age of 18, and those that focused only on health service delivery. We also excluded grey literature, poster abstracts, case reports, reviews, dissertations, books and book chapters, protocols, conference proceedings and presentations, and non-English papers.

### 2.2. Search Strategy

We searched titles and abstracts in CINAHL, Embase, PsycINFO and PubMed databases from inception to April 2020. Key search terms included Indigenous terms used in Canada, Aotearoa (New Zealand) and the United States, and wellbeing and quality of life terms (see Table 1).

### 2.3. Study Selection

**Figure 1:**
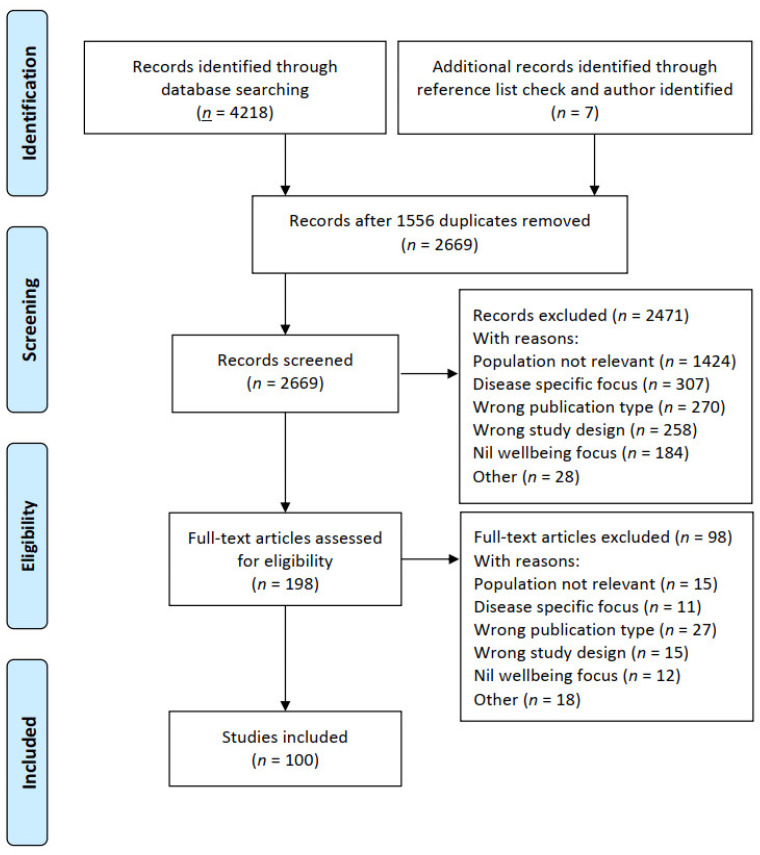
PRISMA schema.

As shown in Figure 1, a total of 4218 papers were identified in the search, with a further six papers identified through hand-searching the reference lists of included papers and relevant earlier review papers (*n* = 6). An additional paper known to the authors but not indexed in the included databases at the time of the search was also included (*n* = 1). After removing duplicates (*n* = 1556), the titles, abstracts, and keywords of 2669 studies were screened against the inclusion criteria using Rayyan online software for systematic reviews [13].

Reasons for exclusion were coded according to a predetermined hierarchy of exclusion reasons (see Appendix A). Four reviewers (AG, KA, GG and KH) screened the title and abstracts of 30 articles with moderate–high consensus between all reviewers. Differences were discussed to improve the accuracy and consistency of further screening. A further 10 percent of papers were double-screened (AG, KA, GG and KH), with very high consensus. The remaining titles and abstracts were then single-screened (AG and KA). Where a consensus could not be reached, the paper was included in the full-text screening phase for further scrutiny. In total, 2471 papers were excluded after title and abstract screening.

AG and KA independently double-screened, all 198 papers and deemed 100 papers eligible for inclusion in the review. The final 100 included papers were imported into NVivo 12 qualitative data analysis software [14] for data extraction and analysis (see Figure 1). Main reasons for exclusion included: population not relevant (*n* = 1439), disease-specific focus (*n* = 318), wrong publication type (*n* = 297), wrong study design (*n* = 273), and nil wellbeing focus (*n* = 196) (see Appendix A).

### 2.4. Data Extraction and Analysis

The following characteristics were extracted from all the included papers by DL then checked by AG for accuracy: publication information (authors, year of publication, paper title, country, region, aim/research question, whether wellbeing was part of the main study aim or broader research question, data collection method, qualitative method, and study setting), and participant information (eligibility, total number of participants, Indigenous group(s) included, and age and sex distribution).

Aspects of wellbeing were extracted using NVivo 12 [14] by AG using the three stages of thematic development recommended by Thomas and Harden for thematic synthesis of qualitative research [15]. Themes were defined as any aspect described in the paper as important to the wellbeing of Indigenous people in Canada, Aotearoa (New Zealand) and the United States. AG extracted the themes for each country separately; analyses were also conducted separately for each country. The data were coded by AG ‘line-by-line’ and then developed into ‘descriptive themes’ that were checked by KA and GG for accuracy and coverage of coding and theme development. The third stage recommended by Thomas and Harden [15] was adapted; Indigenous co-authors from Aotearoa (New Zealand) (EW), Canada (AK), and the United States (MC) were invited to review their respective country’s results to ensure correct interpretation and reporting of the data.

## 3. Results

Across Canada, Aotearoa (New Zealand) and the United States, different words are used in the included papers to describe wellbeing such as wellness and well-being, as well as specific Indigenous language words. How these words are chosen and defined by each Indigenous group is critically important. However, to aid the reader throughout this review, we will respectfully use the word wellbeing, as the majority of the included papers across the three countries used this as their primary terminology to describe Indigenous peoples’ wellbeing.

### 3.1. Paper Characteristics

Of the 100 papers included in our review, 43 were from Canada, 16 were from Aotearoa (New Zealand) and 41 were from the United States (see Supplementary Materials Table S1). In brief, the vast majority of papers were solely qualitative studies (*n* = 91); collected data via face-to-face interviews (*n* = 80); and recruited participants via the community, often with the assistance of a community contact (*n* = 61). Wellbeing was part of the main research aim in 52 papers, with 48 including this as a component of the broader research question. 

Most of the papers included only an Indigenous sample (*n* = 72), 10 included a mixed sample of Indigenous and non-Indigenous participants, and in a further 18 papers, it was unclear if the sample was solely Indigenous or may have included some non-Indigenous peoples. Of those where the sample included both Indigenous and non-Indigenous peoples, we only included the views of those who could be identified as Indigenous. Throughout the results, we have incorporated the country-specific terminology used in the included papers to refer to the Indigenous peoples of their respective countries.

### 3.2. Thematic Synthesis Results

Our team chose to analyse the existing evidence around the wellbeing of Indigenous peoples both within and between the three countries included in this review. While our decision in this regard was guided by practical considerations, we wish to acknowledge that the boundaries of contemporary nation states do not always align with those of Indigenous nations, tribes and groups. To account for the complexities associated with these circumstances, we have also looked at the similarities in wellbeing between countries, which may in part be driven by some of these inconsistencies between Indigenous cultural groups and Western borders.

#### 3.2.1. Canada

Forty-three [16,17,18,19,20,21,22,23,24,25,26,27,28,29,30,31,32,33,34,35,36,37,38,39,40,41,42,43,44,45,46,47,48,49,50,51,52,53,54,55,56,57,58] papers came from Canada; ‘Indigenous peoples in Canada’ is used to collectively report on the aspects of wellbeing as reported in the included papers for First Nations, Métis and Inuit [59]. 

Our analysis revealed seven interrelated aspects of life as being centrally important to the Indigenous peoples in Canada’s wellbeing: Holism/Wholism (*n* = 15 papers); Culture (*n* = 33); Community and Family (*n* = 31); Land and Sea (*n* = 27); Resilience (*n* = 21); Spirituality and Cultural Medicine (*n* = 18); Physical, Mental and Emotional Wellbeing (*n* = 26).

##### Holism/Wholism

The Indigenous peoples in Canada’s conceptualisations of wellbeing as being holistic (includes the whole person—including spiritual, mental, emotional and physical aspects of self) [60] were reported by 15 papers [16,19,20,21,30,32,35,37,42,45,49,51,52,56,58]. Many participants explicitly located the concept of wellbeing within the Medicine Wheel, containing the quadrants: mental, physical, emotional and spiritual [19,30,32,36,49,52,56,58]. Whilst it was recognised that these can be separate dimensions, they were also described as being connected [19,30,32,36,49,56,58]. Some emphasised the need for connection between these quadrants in order to achieve good wellbeing [16,19,35,37,42,45], whilst others described wellbeing as a state of balance across these [19,20,21,42,45,51].

*“In the four components, I try to keep a balance… I think of the four areas, emotional, physical, spiritual and mental.”*

[36]

Several papers emphasised this connection in terms of the individuals being connected with the land and each other [16,19,30,32,35,36,37,42,45,49,52,56]. The quality of each of these complex connections was said to be central in achieving wellbeing.

*“The way any of your parts are treated will affect the whole, and it’s not just the way you treat them but the way that you are treated by the people and systems in your life, and the cultures that you are part of, or that impact you.”*

[19]

##### Culture

Culture was reported as an important aspect of wellbeing for the Indigenous peoples in Canada by 33 papers [16,19,20,21,22,25,26,27,28,29,30,31,32,33,35,36,37,38,39,40,42,43,45,47,48,49,51,52,53,54,55,56,58]. Two key aspects of culture emerged as critical to wellbeing—identity as Indigenous peoples and the importance of one’s Indigenous language.

*“…culture [is] essential for family well-being… [it is a] source of support that ‘bound individuals together’…”*

[28]

*Identity*

Identity was reported by 18 papers as important to the wellbeing of the Indigenous peoples in Canada [19,25,26,30,31,36,38,39,40,43,45,48,49,51,53,54,56,58]. One’s identity as being Indigenous was fundamentally connected to healing and gave a strong sense of pride in being Indigenous, which improved one’s wellbeing [19,25,30,31,36,39,40,45,49,51,53,54,58]. Practicing cultural ways [25,40,43,45,48,53,58], speaking one’s Indigenous language [40,53], hunting and fishing [25,48], and being one with the land [26,31,36,51,56] were reported as important to their wellbeing.

Elders and grandparents said that healing occurs when cultural knowledge that is important to identity is passed down [53]. Identity was derived through connection with land and culture and having a role in family and community [36,49,51,58].

*“…understanding who I am, who I am in terms of my family, my parents and my siblings, uncles, aunts and where I fit in the community, my relationships, aunts, uncles and all of that.”*

[36]

The impact of removal from one’s community and family was reported as negatively influencing some peoples wellbeing. In some papers, it was reported that those adopted or taken into foster care are reconnecting with their cultural identity [19,38,49,53].

“It’s hard because we were raised white…But you know, I’m okay and I’m slowly learning more things about who I am—the connections and the boundaries.”

[53]

*Language*

The importance of one’s Indigenous language, learning and knowing it, was reported by twelve papers [19,22,27,29,33,36,38,40,45,49,53,54]. Language was reported as fundamentally connected to culture, and language was regarded an important means for passing on, preserving and continuing culture, while also bringing a sense of pride, confidence and improved family, community and spiritual connections for the Indigenous peoples in Canada [19,22,28,29,38,40,45,49,53,54,55]. This is not to exclude those without access to language, as culture can and is passed down without it.

*“…we believe that the language is the culture and the culture is the language and the two go hand-in-hand.”*

[49]

##### Community and Family

Family and community was reported by 31 papers as important to the wellbeing of the Indigenous peoples in Canada [17,18,19,20,22,23,24,25,27,28,29,30,31,35,36,37,39,41,42,43,45,46,47,48,49,50,51,53,54,55,57]. Community is broadly defined as those you have connections and communication with, and it is not limited by geographic location. In the included papers, the quality and maintenance of connections and communication with community was described as a central component to one’s wellbeing. Community connection and communication played a significant role in wellbeing for community members [19,22,23,24,25,28,29,30,31,35,36,37,39,42,43,46,47,57], especially when able to find positive and trusting relationships within the community [19,22,29,30,35,36,47]. This was emphasised as important when able to speak freely without fear of judgement, which improved overall wellbeing [19,22,29,30,35,36,47].

*“…talking to one or more others was essential to one’s well-being…talking was identified as the significant component of prevention, intervention, and healing.”*

[35]

Opportunities to be social and attend social engagements was reported as important to wellbeing; being able to connect with community during community events, regular group gatherings, performing cultural activities and sharing meals/resources were all seen to reduce issues associated with isolation and, in turn, build strong relationships [19,22,25,29,31,36,43,46,47]. Where these connections were lacking, either due to distances apart or climate change reducing access to land, participants spoke of a desire to connect to improve wellbeing [23,24,28,31,37,43].

*“…as a marginalized people, [we] have a very big void to address in that area… [people] may not even know they belong to a community. And when you’re isolated like that we all tend to go to the dark side so to speak. A healthy community is a strong community.”*

[19]

Supporting other community members was reported as important to Indigenous peoples in Canada [20,22,24,25,28,29,30,35,36,39,41,45,51,53]. Some participants spoke about needing the support of others [25,30,41], and how providing support boosts wellbeing [22,30,36], while others explained that caring for others can impact wellbeing in complex ways (both in a positive and negative way) [20,28].

*“…looking after children… many of those children are now adults who come to me to tell me I was good to them while they were young. It feels really good inside to know that they have not forgotten me. It’s a rewarding feeling.”*

[22]

An important way that the Indigenous peoples in Canada support each other and improve the wellbeing of the whole community is through the sharing of resources, especially food [24,25,28,29,35,36,39,45]. This highlights the importance of food quality, food security and food sovereignty to the wellbeing of the Indigenous peoples in Canada (these considerations also relate to the *Land and Sea,* and the *Physical, Mental and Emotional Wellbeing* themes).

*“The meat was divided accordingly, nobody was left behind. The men would be up all night carving the meat, and people would come by to pick up their share… The people… were looking after the community.”*

[24]

Some papers reported that the wellbeing of the entire community required cohesion and self-determination, with strong leadership, guidance and teaching from Elders, and collective decision making that prioritised the needs of the community [17,27,28,29,31,35,36,39,45,48,51,54,55,57].

*“Home is living in a [healthy] community where there are elders to undertake (Indigenous) teachings in sharing circles and different ceremonies. This is what makes us who we are. We get our self-identity through communal living and the teachings that go on in the communities.”*

[17]

Family was reported by 20 papers as important to the wellbeing of Indigenous peoples in Canada [17,18,22,23,27,28,29,30,35,36,37,39,42,43,45,47,49,53,54,55], particularly family connection, Elders holding and sharing cultural knowledge within the family and the importance of passing this down through the generations. Connecting with family brought a sense of pride, belonging and security [22,30,35,39,43,53], which, when lost, had a negative impact on wellbeing [17,35,36]. The binding together of families through these connections was often enacted through the family participating in cultural activities together [28,35,39,42,43,47,53].

*“[Family] provided acceptance and a sense of belonging, pride and respect… provid[ing] them with hope, knowing that they can always turn to a family member or friends for support.”*

[30]

*“…culture [is] essential for family well-being… [culture] ‘bound individuals together’ via sharing food, sharing language, hearing Elders’ stories and going out on the land.”*

[28]

The concept of family for Indigenous peoples in Canada is broader than nuclear family structures. For Indigenous peoples, it extends to distant relatives, friends and chosen family (i.e., non-blood-related people they consider family), which provides an individual with a large and diverse support network, and, in turn, fosters and strengthens wellbeing [17,39,47,49].

Elders were reported as being the cultural knowledge holders, and recognition of this was important to wellbeing [18,22,23,28,35,36,37,39,43,45,53,55]. The passing down of cultural knowledge within the family from Elders, grandparents and parents to youth was described as an important aspect of wellbeing [22,27,29,35,36,39,43,45,53,54,55]. The perceived loss of cultural knowledge among today’s young people was seen as concerning for youth’s wellbeing, as well as the wellbeing of future generations.

*“But the times have changed a lot and the young people are now in a place where they don’t really know the old traditional ways but they know the new way more… And it’s due to the fact that the elder’s voices are starting to, you know, diminish…I want the young people to know that. Elders are an important part of their life.”*

[35]

##### Land and Sea

Land and sea was reported by 27 papers as “*vital*” to the wellbeing of the Indigenous peoples in Canada [16,24,25,26,27,28,31,32,35,36,37,39,40,42,43,44,45,46,47,48,49,50,52,53,54,56,57]. The importance of land, encompassing land, sky, sea and animals, is culturally embedded; the land “*is in their bones and in their blood* [31]”.

Specifically, Indigenous peoples in Canada reported the importance of having a spiritual and cultural connection to land [24,26,31,32,36,37,39,40,42,44,46,47,48,49,50,53,54,56] that is deeply rooted in ancestral connections to the regions, giving a sense of peace and belonging that directly improves their mental and emotional wellbeing [24,26,31,32,54,56].

*“We have a connection to these places; our ancestors have occupied this space for thousands of years. The spirit of our people is here. We feel connected to our ancestors in this way.”*

[24]

The relationship between land and people is complex and reciprocal, bringing connection with past and future generations, as well as all of creation [16,25,26,28,31,32,36,37,45,47,48,50,52,54,56,57]. The land provides through food and cultural activities such as hunting and fishing [16,24,25,27,28,35,36,37,39,44,45,46,48,50,54,56,57], and it nourishes the mind and spirit, bringing peace [26,32,37,45,56]. Indigenous people in Canada see their role as more than caretakers or stewards of the land, with a greater purpose of serving all of creation.

*“The respect that we need to show the land and its relatedness to us. We are the land. If the land is sick then it ain’t going to be very long before we’re going to get sick.”*

[54]

The importance of this reciprocity, in the sense of the land being a teacher and the importance of passing on cultural knowledge through the generations, was reported in several papers [24,28,32,35,39,44,46,50,54]. The sense that the teachings of the land have not been lost, but rather need to be reawakened and learnt again, was evident throughout.

*“…nature also becomes a guiding force or spiritual teacher that can help provide a sense of purpose and meaning in one’s life… land as teacher…”*

[32]

Climate change, industry, pollution and technology have all had negative impacts on the land and the Indigenous peoples in Canada’s ability to access it [24,26,31,37,43,44,45,46,47,48,50,52,54,57]. These environmental issues have an impact on wellbeing by creating a barrier to accessing the land, pollution tainting the environment and making cultural foods unsafe to eat, which reduces the wellbeing that is created and maintained through the land [24,26,31,37,43,44,45,46,47,48,50,52,54,57].

*“These rapid [climate] changes were described as disrupting hunting, fishing, foraging, trapping, and traveling to cabins because people were unable to travel regularly (or at all) due to dangerous travel conditions and unpredictable weather patterns.”*

[26]

##### Resilience

Stories of resilience were cited by 21 papers as important to the wellbeing of the Indigenous peoples in Canada, due to the effects of colonisation and how, through resilience, they have been able to protect collective history, culture and build the strength of the community [16,19,22,23,24,28,29,33,35,36,38,41,42,45,48,49,50,53,54,55,57]. Resilience is culturally grounded, including:

*“…culturally distinctive concepts of the person, the importance of collective history, the richness of Aboriginal languages and traditions, and the importance of individual and collective agency and activism”*

(p. 88 [61])

*“Although, there were clear power imbalances, the elders still perceived Aboriginal people to have some agency and power over their existence and cultural practices, resisting the encroaching European and ‘White’ dominance.”*

[33]

Resilience was especially evident in the papers that spoke about residential schools and the lasting impact that these negative experiences had on wellbeing [22,24,29,33,36,38,53,54].

*“…I dealt with all those hard emotional issues and began to build my life as someone with self-esteem… I eventually settled into who I was, my culture, and learned about residential schools and started a path of forgiveness for those that had harmed me.”*

[38]

Resilience was seen in acts of resistance to the rules imposed by residential schools, local authorities and the Church. Such rules were aimed at preventing the Indigenous peoples in Canada from engaging in spiritual and cultural practices [33,53,54], which had severe and ongoing impacts on wellbeing [33,42,50,53,54,57]. Those who defied the rules found this enabled them to hold onto their identity, culture and connections, which was important for rebuilding wellbeing later in life.

*“They’d all take their bundles; their sacred items and they’d go up the river. Way up the river in the secret that’s where they’d do their ceremonies. They would never, ever do it in the community because it was against the law. You went to jail if you were caught doing those things.”*

[54]

Resilience was demonstrated in a number of ways, including the demand for culturally safe services [19,22,23,28,49], and enacting autonomy in the face of pervasive racism and stigma [16,22,23,24,29,38,41,45,49]. The Indigenous peoples in Canada enacted resilience through stories of resistance and activism [16,29,33,38,41,48,50,54]. Resistance and, ultimately, the regaining of self-determination were seen as fundamental to the wellbeing of the whole community [42]. Learning to navigate through “*two worlds* ”(Indigenous/cultural and colonised/Christianised) was reported as important to wellbeing [33,35,36,53,55,57].

*“…[we] try to balance those two worlds… And those [cultural] teachings have to come back in order to know who we are and how to balance ourselves.”*

[36]

Resistance against negative stereotypes and stories about the Indigenous peoples in Canada was said to be central in changing others’ perceptions of them, as well as for the identity and wellbeing of the Indigenous peoples in Canada themselves.

*“I really believe it’s important that our [Aboriginal peoples] story is accurately portrayed. New people to this country, as well as most Canadian citizens, need to know our story, need to understand the impacts of colonization and the residential school system on our cultures, history and languages, and the future impacts these will have for generations to come.”*

[22]

##### Spirituality and Cultural Medicine

The Indigenous peoples in Canada’s spirituality and cultural medicine were reported by 18 papers as important to wellbeing [17,18,19,20,21,22,30,32,33,34,36,42,45,46,53,54,56,58]. 

Cultural medicines, due to their holistic nature and ability to heal spiritually, were important in healing and wellbeing [18,19,34,36,42,45,46,56,58]. This was described as achieving a holistic balance.

*“…traditional healers [are] holistic practitioners, addressing body, mind, and spirit, which [is] different from the Western approach….”*

[34]

Belief in a higher power, referred to as “*the Creator*” and “*Mother Nature*”, was described by some as the foundation to wellbeing [20,22,30,32,46], and was expressed through prayer [22,32,56] and spiritual connection [20,30,46,56]. A number of cultural practices including smudging, sweat lodges, cultural dances and ceremonies, were seen as important to maintaining spiritual and cultural connections [17,30,33,36,42,53,54,56,58].

*“One way to get in tune with the earth is to go to a sweat lodge… You go into the sweat praying and sweating. It is a cleansing and the whole time you are in there, you are praying… You feel so good when you come out of there.”*

[56]

Spirituality improved one’s mental health and gave a sense of place, through important connections to those who have passed and who’s presence is still felt today [22,36].

*“We each have our own souls and our ancestors are in our hearts. I think our ancestors are always with us and we walk with them.”*

[22]

##### Physical, Mental and Emotional Wellbeing

Physical, mental and emotional wellbeing were reported by 26 papers as important to the overall wellbeing of the Indigenous peoples in Canada [17,19,20,21,22,23,26,27,28,29,30,31,32,33,36,38,42,44,46,47,49,52,53,54,56,58].

Physical wellbeing was reported by 15 papers as being important to wellbeing [19,20,21,22,23,26,27,28,30,31,42,44,46,49,52]. This included the need for basic services such as adequate housing, transport and food security [23,27,28,31,42,44,49]. Exercise, diet and lifestyle were all seen as having an impact on wellbeing, especially where there was limited access to cultural foods and activities that often increased activity levels, and where changes in the environment and pollution impacted on diet [19,20,21,22,23,26,30,31,46,49,52]. High food costs, inadequate housing, financial issues and employment all impacted on physical wellbeing in terms of provision of basic needs and maintaining cultural connection [23,27,28,30,31,42,44,52].

*“Land-based, cultural activities are an important component of physical activity….”*

[31]

*“…traditional foods are a lot healthier than the majority of store-bought foods, but due to circumstances such as the changing environment and decreasing numbers of traditional animals, a lot of community members are consuming more store-bought foods.”*

[52]

For older people and those with disabilities, having access to culturally safe services and welcoming places to rest while using these services was seen as important to wellbeing [22,23,42].

*The First Nations older adults also felt that accessibility played a significant role in their ability to age well (safe, accessible, flexible, and affordable transportation in the city). This was particularly the case for the participants who identified as having a disability(ies).”*

[23]

Mental and emotional wellbeing was reported as important to the overall wellbeing of the Indigenous peoples in Canada in 19 papers [17,19,20,21,26,29,30,31,32,33,36,38,47,49,51,53,54,56,58]. Understandings of mental and emotional wellbeing were intertwined with the Medicine Wheel, where all aspects of the wheel need to be in balance in order to achieve and maintain mental and emotional wellbeing [19,49,51,58]. Connecting to culture was seen as critical to maintaining mental and emotional wellbeing, including connecting spiritually and to the land [26,30,31,32,36,47,49,51,54,56].

*“So mental health isn’t just mental health, it’s spiritual health, physical health and emotional health as well.”*

[19]

Relationships with family and the community, including supporting each other, were seen as important to maintaining and building healthy mental and emotional wellbeing [19,30,36,47,49,51].

*“mental health and well-being is not a new concept. However, given the history of colonization and especially the residential school legacy, it is essential to understand the increased significance of relationships on contemporary Indigenous peoples’ mental health and well-being.”*

[30]

Trauma was a recurring theme, especially associated with residential schools and abuse, manifesting intergenerationally, as pain was passed down through the generations [19,29,30,31,36,38,49,53]. “*Dealing*” with the trauma and talking through their pain and their emotions were seen as means to regain wellbeing [19,20,21,51,58].

*“You can deal with intergenerational trauma as well by acknowledging it, realizing it, respecting it, learning from it, forgiving it… the biggest gift I ever gave myself, was really doing that hard trauma work.”*

[19]

Maintaining mental wellbeing required being actively employed and not being idle [20,21,36,47].

*“Respondents believed that not being gainfully engaged in anything significantly impacts the mind may eventually result in mental disorders. They said unemployment creates idleness and redundancy that negatively affects mood and physical wellbeing.”*

[36]

#### 3.2.2. Aotearoa (New Zealand)

There were 16 papers relating to the wellbeing of the Indigenous peoples in Aotearoa (New Zealand), the Māori people [62,63,64,65,66,67,68,69,70,71,72,73,74,75,76,77]. Our analysis revealed five interrelated aspects of life as being centrally important to Māori wellbeing: *Māoritanga* (Māori identity; *n* = 9 papers); *tikanga* (Māori customs; *n* = 12); *kotahitanga* (togetherness and connection; *n* = 13); *whakapapa* (importance of genealogies; *n* = 15); *wairuatanga* (spirituality; *n* = 8).

##### Māoritanga—Identity

*Māoritanga* was cited by nine papers as having a central role in Māori wellbeing [64,66,69,70,71,72,74,75,77]. It can be translated as meaning identity, as it encompasses Māori culture, practices and beliefs [78]. This broad reaching concept was described, not only as a vital component of wellbeing, but also as an overarching concept that connected and united other aspects of life that emerged as important to Māori wellbeing. *Māoritanga* is cultivated and strengthened through connections with Māori culture, Māori language, connections to the land and whānau (family).

*“A collectivist approach to the men’s sense of self is lived through connections with whanau.”*

[64]

Culture is an important ‘living’ factor to *Māoritanga*, both for the individual and the collective [66,71,72,77]. One paper identified the importance of learning Māori language to Māori women in reclaiming cultural identity [72]. Participating in cultural activities, such as *Kapa Haka* (Māori singing and dancing), was described as adding to *Māoritanga* of Māori as a collective [72]. This was also true of Māori responsibility to attend sites of significance to the *iwi* (tribe) [70]. These aspects of participating in Māori culture and fostering *Māoritanga* were put forward as the basis for building wellbeing among Māori people.

*“Māori identity and culture includes obligations and responsibilities to attend activities and sites connected not only to their tribe, but also their family identity.”*

[70]

*Māoritanga* also incorporates Māori connection to the land and guardianship of it [69,70]. The central importance of land to Māori wellbeing was illustrated by the Māori custom of using geographical land features such as *maunga* (mountains), *awa* (rivers) and *moana* (oceans) to identify themselves as individuals within the collectives of the *hapu* (sub-tribe) and *iwi* (tribe) [69].

*“Māori identity is linked to the earth by a sense of belonging to the land, being part of the land and being bonded together with the land.”*

[69]

The forced assimilation of Māori when *Pākehā* (Europeans) came to Aotearoa (New Zealand) was seen as a source of conflict [71]. Reconciliation was described as fraught and the act of forgiveness for the past was described as involving further damage to *Māoritanga* [71].

##### Tikanga—Māori Customs

Twelve papers reported *Tikanga* as being important to Māori wellbeing [62,63,64,65,67,69,70,71,72,73,74,75]. *Tikanga* can be translated to mean Māori customs, practices and ways of doing things that are deeply embedded in Māori culture [75,78]. The papers reported four parts of *tikanga*: (a) *pūrakau* (ancient stories or legends) and *whakatauki* (proverbs); (b) *iwi* (tribe) control, self-determination and autonomy; (c) *manaakitanga*—to extend *aroha* (love/compassion) and *mana* (respect/power) to others; and (d) Colonisation and Māori–*Pākehā* (Europeans) relations. These papers also spoke of the disconnect of Māori from *tikanga* caused by the colonisation by *Pākehā* (Europeans) within Aotearoa (New Zealand).

*Pūrakau (ancient story or legend) and whakatauki (proverb)*

*Pūrakau* and *whakatauki* were reported in five papers as important to the wellbeing of Māori [62,65,67,69,74]. *Pūrakau* and *whakatauki* are the narrative tools with which Māori pass down cultural wisdom and knowledge [78]. A sense of pride was associated with passing down knowledge through the generations [62,65,67,74] as was instilling pride in others, creating connections between people through sharing and knowing family history [62,65,67,74]. Both the content of these stories and proverbs and also, the act of sharing them and passing down this knowledge, are intrinsic to Māori culture and maintain a strong connection to the land [65,69]. The sharing of knowledge in this way was described as locating Māori, giving a sense of place and identity for Māori now and future generations.

“One of Winiata’s aspirations is to ensure he passes on his practical skills and knowledge of ‘the bush’ to younger generations, most of whom now live in urban centres… [this] can ensure younger whānau have an embodied and emplaced experience of their belonging in this place as their tūrangawaewae (place of belonging).”

[74]

*Iwi (tribal) control, self-determination and autonomy*

*Iwi* control, self-determination and autonomy were described as important to the wellbeing of Māori [67,72,73], not only to the individual, but also to the collective [73]. Individuals and the collective need to be able to exercise self-determination, which results in agency and autonomy over Māori people’s lives, including individuals and the whole *iwi* [67,72,73].

*“The ability for Māori leaders to not only be self-governing in their behaviour, but to develop others’ autonomy and self-determination, triggered satisfaction and consequently enhanced well-being.”*

[73]

Two papers highlighted the importance of *iwi* sovereignty over decision making of service provision and policy for Māori communities [67,73]. This was exemplified in the context of housing and homelessness, where the value of addressing issues at a community level was highlighted as imperative to promoting wellbeing of all Māori [67,73].

*“…a duty of iwi to act on their position as “sovereign iwi nations” was identified by several of the respondents… public policy discourse around iwi taking charge of the responses to the needs of their populations.”*

[67]

*Manaakitanga—to extend aroha (love/compassion) and mana (respect/power) to others*

Seven papers spoke about the importance of *manaakitanga* to Māori wellbeing [62,64,67,71,72,74,75]. Relationships and social networks were seen as invaluable to Māori wellbeing as they facilitated sharing of important resources, such as food, income, and health care [64]. A fundamental Māori value that underpins the provision of this support is *manaakitanga*, which represents the extension of *aroha* (love/compassion) and *mana* (respect/power) to others [62,67,71,75]. This custom has practical applications and implications for Māori, by encouraging the sharing of resources critical to wellbeing. For example, in many settings, the food security of individuals, *whānau* (family) and the whole *iwi* (tribe) is underpinned by *manaakitanga* [62]. 

*Manaakitanga* is also important to bringing and maintaining wellbeing and inner balance through the act of serving and providing for others [71,75]. This reciprocal relationship, whereby the giver and receiver both benefit, is a deeply spiritual aspect of *manaakitanga* whereby the “*purpose of life is to serve others* [75]”.

*“Reciprocity is at the heart of manaakitanga, and rests upon a precept that being of service enhances the mana (authority/power) of others… Manaakitanga transforms mana through acts of generosity that enhances all, produces well-being… uplifting the mana of others in turn nourishes one’s own mana.”*

[75]

*Colonisation and Māori–Pākehā (European) relations*

Colonisation and Māori–*Pākehā* relations were reported by seven papers as having a negative effect on Māori wellbeing [63,67,69,70,71,72,75]. While colonisation may have affected other aspects of Māori wellbeing, the included papers focused on the impact of colonisation on *tikanga* (Māori customs), principally through the loss of autonomy and *iwi* (tribe) control.

*“…participants described barriers to participation in planning and active exclusion of Māori from decision making; for example, actions of the local council were identified as symptomatic of ongoing colonisation and oppression. A lack of voice in decision making…”*

[70]

This disempowerment was acutely felt by Māori who saw this *Pākehā* control as having a direct influence on *Māoritanga* (identity), wellbeing and physical health. Conflicts between Māori and *Pākehā* were evident through interpersonal racism and institutional racism within the systems of health and politics that create significant barriers for Māori [71,72].

*“Participants conveyed feelings of grief and shame at being culturally disenfranchised… As long as Pākehā [colonisers] or politics are always running these [health care systems], I don’t know if we’re going to get any better.”*

[63]

##### Kotahitanga—Togetherness and Connection

The importance of *kotahitanga* to Māori wellbeing was reported by 13 papers [62,63,64,65,66,69,70,71,72,73,74,75,76]. *Kotahitanga* encompasses the Māori concept of togetherness and connection [78]. The included papers referred extensively to different aspects of *kotahitanga* as they relate to wellbeing, most commonly via Māori relationships to the physical environment through *kaitiakitanga* (guardianship of land), with *whānau*, *hapu* and *iwi* (*whakapapa*—genealogies), and relationships in general (*whanaungatanga*). While, within Māori ideology, *whakapapa* (genealogies) is encompassed by *kotahitanga*, due to the significant importance of this Māori aspect to wellbeing, and its extensive coverage across the papers, it is described as a separate theme.

*Kaitiakitanga—guardianship of the land*

The importance of *kaitiakitanga* to the wellbeing of Māori was reported by nine papers [62,63,64,66,69,70,74,75,76]. *Kaitiakitanga* is the Māori custom that encompasses the duty of being guardians of the land [78]. Some papers described *kaitiakitanga* as central to *Māoritanga* (identity) [70] with Māori health and wellbeing being directly related to the health and wellbeing of the land [63,69,74,75,76].

Fulfilling *kaitiakitanga* is culturally embedded [63,75]. Māori connection to the physical environment is spiritually based and Māori consider themselves as being “*of*” the environment [63,69,74,75], which, in turn, contributes to *Māoritanga* (identity) [66]. This sense of spiritual connection was described as unifying the individual in the environment, which was a central quality underpinning wellbeing.

*“…[We have] a spiritual connectedness… with the land and the environment in which I live… It just makes me feel whole, and complete… my maunga (mountain) and… my awa (river)… directly affect my mental health and my physical health….”*

[63]

In one paper, Māori healers outlined how transgressions against the land directly affect the health of the individual [69]. The intricate symbiotic relationship between Māori and the land is described as a direct relationship between the wellbeing of both [76]. This two-way relationship gave Māori the freedom to live off the land and stay away from larger cities, which improved overall wellbeing [64].

*“…also understanding the relationship between the health and wellbeing of ourselves as people and the health and wellbeing of our lands, of our rivers, of our oceans, of our mountains, of all of the environment. So, I think there are definite links between the health and wellbeing of people and the health and wellbeing of our environment.”*

[76]

*Kaitiakitanga* was also described as important to Māori relationships with people, fostering stronger ties between people and building *Māoritanga* (identity) [64]. When Māori are displaced from the land, they reported feeling grief, disenfranchisement and cultural displacement [63], and also the undermining of the Māori collective identity [64].

*Whanaungatanga–relationships, kinships and sense of family connection*

The importance of *whanaungatanga* to Māori wellbeing was reported by six papers [65,71,72,73,74,75]. *Whanaungatanga* represents kinships, both formal and informal, that are created through shared experiences [78]. Relationships, especially with significant others, were seen as having an impact on Māori wellbeing via *Māoritanga* (identity), with one paper citing the need to nurture *whakapapa* (genealogies) relationships in order to maintain the interconnections of being Māori [74]. 

The concept of *whanaungatanga* recognises the importance of building healthy relationships with others outside of *whānau* (family) [65,75]. The act of supporting the wellbeing of other people is seen as an important part of establishing and maintaining healthy *whanaungatanga*.

*“I always think of whanaungatanga [relationships], manaakitanga [practising respect and kindness] and I think I’m here for the well-being of the people.”*

[75]

*Whanaungatanga* was described as being built and maintained through *tauutuutu* (reciprocity) and *mana* (respect), which, in turn, were said to produce positive impacts on the wellbeing of the individual practicing these customs [71,73,75]. 

##### Whakapapa—The Importance of Genealogies

Fifteen papers described *whakapapa* as important to the wellbeing of Māori [62,63,64,65,66,67,69,70,71,72,73,74,75,76,77]. *Whakapapa* encompasses the “*interconnectedness between people, places, and events over time*” [71] and is a source of Māori identity that is found within genealogies [71,74]. This important connection brings the past into the present, which establishes Māori peoples’ standing within *whakapapa* and *whānau* (family), and physical place on the land (*turangawaewae*) [64,67,69,71,74,75]. 

*Whānau—Family*

The importance of *whānau*, the connection to *hapu* (sub-tribe) and *iwi* (tribe), *whānau* support, and how this connection directly affects Māori wellbeing was described in twelve papers [62,63,64,66,69,70,72,73,74,75,76,77]. Māori involvement with *whānau*, *hapu* (sub-tribe) and *iwi* (tribe), was outlined as the primary reason for living and establishing an individual’s role and *Māoritanga* (identity) [63,64,66,73,74]. When displaced from *whānau*, Māori were left feeling disconnected and lacking a sense of place (*turangawaewae*) [63], thus significantly reducing wellbeing. 

Connection to *whānau* plays an important role in the wellbeing of the individual with healers citing sometimes it is necessary for *whānau* involvement in order to achieve spiritual and physical healing of Māori [69]. This strong connection between *whānau* wellbeing and an individual’s wellbeing was evident throughout five of the included papers [63,64,69,72,76] and was clearly stated by one paper [72].

*“If my whānau are not well, I am not well.”*

[72]

Support from *whānau*, *hapu* (sub-tribe) and *iwi* (tribe) was reported as providing differing benefits which improved Māori wellbeing [62,64,70,72,73,74,76]. Māori provided each other with material support [62,64,70,74,76], spiritual support [76], nurturing environments and childcare [74,76], and importantly, a sense of hope for the future [64,73]. 

The strong interwoven connections within *whānau* were outlined as an important concept for those in decision making roles relating to Māori. Māori decision making often occurs at an important meeting place that Māori use to facilitate connections, the *marae* (cultural meeting house) [70,73,74]. The *marae* and the connections that occur in this place were identified as bringing Māori strength and cohesion [74].

The importance of physical health in the wellbeing of Māori was only spoken of in the context of *whānau* connection, as it was seen as an enabler of connection rather than important for wellbeing in its own right [62,63].

*“…not saying that I’m unhappy now but I think if I’d accomplished that [weight-loss]… it would mean I’d be able to just do more with my daughter and my partner…”*

[63]

Three papers spoke specifically about the importance of *kaumatua* (elders) and the vital role they play in the wellbeing of the whole *whānau*, *hapu* (sub-tribe) and *iwi* (tribe) as pillars of tradition and living links to *whakapapa* (genealogies) [70,72,74].

*“The presence of elders at hui and other events was highlighted as particularly critical; without their expertise, there was a risk of adverse impacts on the wellbeing of the whole family, including the loss of tikanga (procedures) and kawa (ceremonial etiquette) knowledge.”*

[70]

*Turangawaewae–sense of place*

*Turangawaewae* was described in five papers as important to the wellbeing of Māori [64,65,69,73,74]. *Turangawaewae* is the concept of having a cultural place to stand [74]; this place is established through long held positionings of *whakapapa* (genealogies) [64]. This sense of place is seen as a core component to *Māoritanga* (identity) and keeping the connection of *whānau* (family) strong and alive [64,65,69,74]. Belonging to a *turangawaewae* was described as maintaining *whakapapa* (genealogies) and instilling Māori pride [64,65,74].

*“I belong here. I can stand here without challenge. My ancestors stood here before me. My children will stand tall here.”*

[74]

##### Wairuatanga—Spirituality

*Wairuatanga* was described in eight papers as important to the wellbeing of Māori [63,66,68,69,72,75,76,77]. *Wairuatanga* represents Māori spirituality, which was seen as vital to Māori wellbeing, keeping individuals connected to Māori culture and beliefs and fostering *Māoritanga* (identity) and a sense of belonging [63,66,69,72,75,76]. These papers highlighted the importance of *Rongoa* (Māori medicine), *karakia* (prayers) *waiata* (songs), and *tohu* (signs) as components of *wairuatanga* (spirituality) that impact on wellbeing [63,66,68,69,72,75,76,77]. 

*Rongoa—Māori Medicine*

An important practice of *wairuatanga* (spirituality), which was reported by seven papers, was *rongoa* [63,66,68,69,75,76,77]. Underpinning *rongoa* is the holistic understanding of health, which emphasises the need for balance in all things in order to feel well [63,66,68,69,76,77].

*“…so all those things, spirituality, physical health, mental health, family health, I think are equal in Māoritanga… if ones out then the rest is out.”*

[76]

Views on wellbeing were purposively sampled from Māori healers in one paper. The authors built a model of what this holistic view on health looks like, using a star to show the interconnections between *hinengaro* (mind), *tinana* (body), *wairua* (spirit), *whakapapa* (genealogies) and *whenua* (land) [69]. *Rongoa* healers explained that the individual is made up of the mind, body and spirit, and when there is disharmony or disconnect, the individual becomes unwell [68]. They emphasised the importance of using this model within the health system to better serve the needs of Māori people [69,77], and Māori people agreed this has a direct effect on wellbeing [77]. 

*Karakia (prayer) waiata (songs), and tohu (signs)*

The importance of *karakia*, *waiata*, and *tohu* to the wellbeing of Māori was reported in five papers [63,66,69,72,76]. Participating in *karakia* and *waiata* was seen as offering Māori a sense of safety, protection and connecting to Māori culture and traditions, which was said to build resilience [66,72]. *Tohu* were spoken of as providing important guidance for Māori people [69]. *Tohu* were related to animals and the natural environment and were important due to strong connections with the environment [69]. Further, these papers described the importance of drawing on these spiritual practices during times of hardship and illness, which helped the whole *whānau* (family) maintain wellbeing [76].

*“…[when] it doesn’t happen [Indigenous custom] it makes me feel uneasy, like something’s not right… they [non-Indigenous people] didn’t do a karakia (Indigenous prayer/incantation)… and that made me feel different….”*

[63]

#### 3.2.3. United States

Of the included studies, 41 [79,80,81,82,83,84,85,86,87,88,89,90,91,92,93,94,95,96,97,98,99,100,101,102,103,104,105,106,107,108,109,110,111,112,113,114,115,116,117,118,119] papers related to the wellbeing of Indigenous peoples in the United States. The United States comprises of different Indigenous groups (American Indians, Alaska Natives and Native Hawaiians) [2,120], with unique cultures and languages. The biggest group is American Indians and Alaska Natives, who are not only a racial designation, but a legal one [120]. To date, 574 Tribes and Alaska Villages are federally recognised by the United States government as sovereign nations and, therefore, governmental entities [120]. Tribal sovereignty and self-determination, as manifested in Tribal governments, are the basis of Tribal and community culture and life, and underscore all aspects of wellbeing. 

Our analysis of the included papers revealed seven interrelated aspects of life as being centrally important to the wellbeing of Indigenous peoples in the United States: Holism (*n* = 10 papers); Culture (*n* = 30); Spirituality and Cultural Medicine (*n* = 28); Tribe/Community and Family (*n* = 29); Land, Sea and Subsistence-based living (*n* = 28); Resilience (*n* = 21); Basic Needs (*n* = 17).

##### Holism

The holistic nature of wellbeing was reported by ten papers [87,94,95,99,101,107,108,110,112,117]. Wellbeing among the Indigenous peoples in the United States is a holistic concept placing value on the integration of physical, mental, emotional and spiritual health [87,94,95,101,108,110,112,117], with some explicitly locating the concept of wellbeing within the conceptualisation of the Medicine Wheel that contain the quadrants: mental, physical, emotional and spiritual [87,95,99].

*“…[wellbeing] is the full integration of the physical, mental, emotional, cultural, and spiritual facets of a person….”*

[108]

In order to realise “*full integration*”, ten papers reported the importance of finding balance [87,94,101,107,110,117], which could be maintained or achieved through strong spiritual and relational connections [95,101,107,112,117], and maintaining a sense of cultural identity [107].

*“wellness is the ability when you get knocked off or you feel out of alignment, it’s the process of coming back to alignment. It’s the process of rebalancing.”*

[101]

##### Culture

The importance of culture to the wellbeing of the Indigenous peoples in the United States was reported by 30 papers [79,80,81,83,85,86,88,90,91,92,93,94,95,96,97,98,99,101,103,105,106,107,111,113,114,115,116,117,118,119]. Culture confers Indigenous peoples in the United States a sense of identity [94], pride [94,114] and strength [88]. 

*Cultural Preservation and Cultural Pride*

Different aspects of cultural preservation and cultural pride dominated this theme, with 29 papers reporting its importance to the wellbeing of the Indigenous peoples in the United States [79,80,81,83,85,86,88,90,91,92,93,94,95,97,98,99,101,103,105,106,107,111,113,114,115,116,117,118,119].

Impacting on the strong desire for cultural preservation was the impact of acculturation (assimilation into the dominant culture) and the consequent relationship disharmony [86,92,115], substance abuse [86,92,115,116] and movement away from cultural and healthy practices [92,103,117]. This loss of culture has left negative imprints across generations, resulting in generational trauma [88,94,95,115,116,117] and a loss of identity [80,92,94,97,101,115,116,117].

*“[through attempts to] Christianize and civilize and assimilate our people, we lost a lot. There’s some generations where some of our people weren’t able to learn the language, weren’t able to learn a lot of things about who we were, our traditional ways… That’s where we lost many of our values and cultural ways… pretty much forced into the assimilation.”*

[92]

Enculturation (acquisition into an individual’s own culture) was described in 17 papers as important to overcoming acculturation, which confer a renewed sense of pride and identity [83,86,88,90,91,92,94,97,98,99,101,107,111,114,115,116,119]. It was reported as being a protective factor of wellbeing [83,90,92,94,99,115,116], bringing cultural healing [90,91,92,101,107,111,115,119].

*“reawakening of our people… to not just understand, but value what we know as Native people, and how that’s instrumental to where we want to go in the future… [thus] strengthening the wellness of the individuals [and] community wellness.”*

[94]

Passing down cultural knowledge through storytelling, mostly by Elders, was reported by 19 papers as important to cultural preservation and wellbeing [79,80,81,83,86,88,91,95,97,99,101,105,106,107,111,113,114,116,118].

*“The Elders… teaching [the youth] traditional values and lifestyles and incorporating Western technology with subsistence activities, all of which contribute to their emotional well-being.”*

[106]

*Language*

The importance of one’s Indigenous language to the wellbeing of Indigenous peoples in the United States was reported by eleven papers [79,80,85,86,92,94,97,98,101,107,116]. Acculturation that followed colonisation saw the progressive loss of Indigenous languages in the United States over time, resulting in grief and concern [80,85,86,92,97,98,101,107,116].

*“When asked why language is so important Alayna replied, ‘it’s really important to our overall health and well-being. It has everything to do with who we are and we’re getting further and further away from that.’…”*

[101]

*“I feel sad because I don’t speak Navajo, I know that I lost something.”*

[86]

*Physical Activity*

Physical activity was reported as important to the wellbeing of Indigenous peoples in the United States in the context of culture (including Tribe/community) by seven papers [79,80,94,96,106,118,119]. Conversely, the desire to be more physically active was proposed through cultural activities to increase cultural engagement.

*“Even moderate exercise, such as staying busy in the community or engaging in subsistence activities, helps improve quality of life, both mentally and physically….”*

[106]

##### Spirituality and Cultural Medicine 

Spirituality and Cultural Medicine were reported as important to the wellbeing of Indigenous peoples in the United States by 28 papers [80,81,82,85,86,87,89,90,91,92,93,94,95,97,99,100,101,104,106,107,108,110,111,112,113,117,118,119]. Spirituality is interwoven throughout life [95,104,106,111,112], forming identity [95,112], providing guidance [100,106,112,117], and for coping with stressors that impact wellbeing [81,90,92,104,106,117].

*“[United States Indigenous Peoples’] emphasized the belief that the path to true wellness and health implies matters of not only physical well-being, but functions of spiritual and mental health as well. Inherent in this idea of holistic balance is that traditional medicine is one of the primary pathways to restoring the imbalance….”*

[110]

*Higher Power or Energy*

The belief and connection to a higher power, or energy, were reported by 14 papers as important to the wellbeing of Indigenous peoples in the United States [80,85,86,87,91,92,93,94,95,99,106,107,111,113]. These powerful and spiritual entities were described as providing rules to live by in order to maintain wellbeing [80,85,87]. In return for abiding by these rules, these spiritual entities provided healing and resources (food, water and medicines) [80,85,87,93,94,107,111,113], and both mental and spiritual balance (e.g., the Medicine Wheel described earlier) [80,91,92,95,99,106,107]. The connection to a higher power was strengthened and maintained through respect [80,85,86,113], cultural ceremonies [80,91,92,94,107,111] and Indigenous languages [94].

*“The Holy People are understood to play a crucial role in the efficacy of the ceremony and in maintaining one’s health and well-being throughout life.”*

[107]

*“Ceremony and language remind people of ‘how all is a part of me and we are all a part of this,’ including nature and connection to higher powers, and how the continual work to ‘restore balance’ ensures the wellness of all parts.”*

[94]

*Cultural Medicine*

Cultural medicines, including ceremonies and dances, were reported as important to the wellbeing of Indigenous peoples in the United States by 19 papers [80,81,82,85,86,87,89,91,93,94,95,97,99,101,107,108,110,111,119]. 

Ceremonies were reported as important due to their use in Cultural Medicine, connecting and reconnecting to each other, and giving a sense of identity (particularly when displaced from home) [80,82,86,87,89,91,93,94,95,97,99,101,107,111]. These ceremonies were primarily used for balance, purification, prevention of illness, healing, memorialising the dead, enhancing spiritual development and for connecting with the higher power [80,82,86,87,89,91,93,94,97,101,107,111]. They are sacred and often include prayers, dances, drums, specific medicinal herbs and important people in the process (medicine men and women, clan aunts and uncles, and family members) [80,82,86,89,91,97,101,107,111]. However, these ceremonies can be performed by individual people when problems arise in their lives [80,89].

*“After our community performed this ritual we felt a difference among each other and in our homes and lives. We all felt lighter and happier. We smiled at each other more easily and things got better for our young people. The ritual brought us together and together we were stronger.”*

[80]

Cultural dances were reported as balancing the four aspects of wellbeing [108], giving Indigenous peoples in the United States renewed strength and pride [101]. They were used as a means to connect with ancestors and a higher power, by both asking and thanking them for resources [80,86].

*“I see a really great relationship between hula and health in all aspects of health, not just physical health, but mental and emotional health, spiritual health…”*

[108] Tribe/Community and Family

Community and family were reported as important to the wellbeing of Indigenous peoples in the United States by 29 papers [79,80,81,83,84,86,87,88,89,90,95,96,97,98,99,100,101,103,104,105,106,107,112,113,114,116,117,118,119].

Tribe/Community was reported as important to the wellbeing of Indigenous peoples in the United States by 26 papers [79,80,81,83,86,88,89,90,96,97,98,99,100,101,103,104,105,106,107,112,113,114,116,117,118,119]. The concept of Tribe/community is broad, including kin relations, clans, Tribal/community relationships at school, work, male and female, elders and others [80,120]. 

Connection to Tribe/community, which included having Tribe/community support and safety, is an important aspect of wellbeing [79,80,86,89,90,96,97,99,100,101,103,104,105,107,112,113,114,116,117,118,119]. Indigenous Tribes/communities are founded on the differing customs, practices and values held by each group [80,113,116]. Tribe/community connections are strengthened when Tribes/communities come together, support each other, and have a Tribal/community role [79,80,89,101,103,104,105,107,113,114,116,117,118,119]; this improves wellbeing through a sense of belonging and identity [89,97,99,101,103,105,107,112,113,114,116,118,119]. Connecting to Tribe/community resulted in strong social supports that enabled healthier living [86,96,103,114]. Safety within the Tribe/community was important to wellbeing [103,105,113,117], and lack of Tribe/community support and connection was seen to have a negative impact on wellbeing [90,100,117].

*“Relationships provide a connection within community as well as an avenue through which to develop identity, both are important concepts….”*

[97]

Elders in the Tribe/community were reported as important to the wellbeing of Indigenous peoples in the United States by 15 papers [79,80,81,83,89,97,98,99,104,105,106,107,112,113,119]. Elders were seen as the knowledge holders who encompassed cultural ways with wisdom [79,80,81,83,97,98,99,105,119]. The reciprocal relationship of knowledge-giver and knowledge-receiver, including respect, improved wellbeing for both individuals and the Tribe/community [80,81,83,97,98,104,105,106,119]. Tribe/community values, often instilled by Elders, were seen as important to Tribe/community wellbeing and the individual, especially the value of non-violence [80,81,83,89,97,99,106,107,112,113,119].

*“kūpuna (Elders)… pass down culture, religion, values, in the right way to the next generation… kūpuna looks to the past and future. Kūpuna are the ones with the knowledge—wisdom. They respect Hawaiian values and deserve respect.”*

[81]

Family, including family connection, was reported by 24 papers as important to the wellbeing of Indigenous peoples in the United States [80,81,83,84,86,87,88,89,90,95,96,97,99,101,103,104,105,106,107,112,113,114,117,118]. Family “*is an active kinship system inclusive of parents, children, cousins, aunties, uncles, and grandparents*”, and can include “*close, trusted friends*”, which is an important distinction for Indigenous peoples in the United States when compared to the greater emphasis on the nuclear family in Western cultures [87,88,95,97].

Family connection was seen as essential to passing down cultural knowledge and how to live in the colonised world [80,84,86,88,89,96,97,101,105,112,114,118]. This sense of family connection went beyond the physical, to strong spiritual connections to ancestors and future generations, and this acted as a guide in the present [89,101,112,113].

*“…familial relationships and spending time together was a large contributor to many participants’ definition of health and wellness.”*

[118]

Family support was reported as important to the wellbeing of Indigenous peoples in the United States by 14 papers [81,83,86,87,88,89,90,96,99,105,106,107,113,118]. “*Family comes first*” [81] was a notion that emphasised that supporting each other was expected [81,86,87,88,99,105]. While supporting family was seen as important to wellbeing both emotionally and physically [81,83,86,89,90,96,99,105,106,107,113,118], others found this taxing on personal wellbeing due to increased financial costs and the inability to perform self-care [81,86,87,88,96].

*“Participants cited family relationships as integral to their health and wellness, as well as a source of ongoing stress.”*

[96]

##### Land, Sea and Subsistence-Based Living

The land and sea, including subsistence-based living, was reported as important to the wellbeing of Indigenous peoples in the United States by 28 papers [79,80,81,84,85,86,87,91,93,94,96,97,99,100,101,102,104,105,106,107,109,112,113,114,116,117,118,119]. 

*Connection*

Indigenous peoples in the United States’ connection to land and sea was reported by 13 papers as important to wellbeing [80,85,91,93,94,96,101,107,112,113,114,117,118]. This deep connection was felt through a sense of identity and a sense of belonging, with the land and sea providing connection to ancestors and each other [80,91,93,94,96,101,107,113,114,117,118].

*“When we think about a healthy community…it includes… love of our natural world… and recogn(izing) our deep connection to it.”*

[94]

*“…I am… a spiritual being connected to land, mountain, sea, especially the ocean….”*

[113]

*Sacred Provider*

The land and sea/water are sacred to Indigenous peoples in the United States [91]; it provides for them through subsistence-based living [80,84,85,104,105,106,116,119] and through their spiritual connections to land [91,107,112,113], as well as providing healing and protection [80,85,91,107,112,113,119]. When displaced from their varying homelands, they become unwell, both physically and mentally [91,93,101,113,114,117]. In order to maintain connection, the land and sea must be cared for and respected [80,85,99,112,113,119].

*‘‘Health comes from the land, and how we take care of the people and the land.”*

[85]

Subsistence-based living can be defined as “*…the traditional economy, living on the land and with the land, that brings meaning to Aboriginal peoples*” (Simon Brascoupé) [121]. Subsistence-based living, including “*living off the land*” [84] and cultural foods, was reported as important to the wellbeing of Indigenous peoples in the United States by 18 papers [79,80,81,84,85,86,87,94,97,100,102,104,105,106,112,116,118,119]. Farming, hunting and gathering of foods brought families and Tribes/community together, giving them a sense of belonging and connection [80,84,104]. It also relieved the financial strain on households, by providing foods and cultural medicines [84,105,116]. It was reported that the movement away from subsistence-based living resulted in suffering and ill-health in the Tribe/community [80,81,85,86,94,100,106,112,116,119].

*“Many participants expressed that this subsistence lifestyle is at the core of wellness for Yup’ik people, frequently referring to it as ‘the lifestyle’ or ‘the way of life’.”*

[119]

Cultural foods were reported as important to the wellbeing of Indigenous peoples in the United States [79,80,81,86,87,94,97,100,102,105,106,112,118,119]. Cultural foods were seen as nutritionally superior to modern Western foods, sustaining health in the past and today [79,81,86,94,100,106,119]. Some foods were considered especially important due to their medicinal and spiritual properties [81,94,102,119]. Cultural foods were seen to provide connection socially to each other, and culturally to home, especially when individuals were displaced [94,102,112,118].

*“Consistent with this emphasis on the subsistence lifestyle for health and wellness, participants in all focus groups remarked on the superiority of traditional natural foods over store-bought food that is processed and imported. Participants described how native foods provide better nutrition, better taste, keep hunger satiated for a longer time and increase physical health.”*

[119]

##### Resilience

Resilience, and the consequent adaptation and need for autonomy, was reported by 21 papers as important to the wellbeing of Indigenous peoples in the United States, especially in the context of living between two worlds (pre-colonisation and post-colonisation) [79,80,82,86,89,90,91,95,96,97,98,99,101,103,104,106,107,110,113,116,117].

*“When probed on what it was that brought tears to her eyes, she added, ‘I think it’s just knowing what we went through and that we’re still trying to keep our culture and still live in the modern way that we have to’.”*

[86]

There were mixed views on the combining of both cultural spiritual beliefs with Christian ones, with some believing this improved wellbeing and others expressing a need to go back to solely cultural spiritual values [82,86,91,95]. Indigenous peoples in the United States found strength through cultural values and practices, while navigating the Western world [101,107]. Colonisation and resultant acculturation brought conflict in families trying to co-exist, guilt and loss of identity in not knowing culture, and was attributed to negative behaviours in the Tribe/community, ultimately impacting on wellbeing [97,101,116,117].

Having autonomy, adapting to the Western world, and building resilience was reported by 13 papers as important to the wellbeing of Indigenous peoples in the United States [79,86,89,90,96,98,99,101,103,104,113,116,117]. Autonomy was achieved through building resilience and adapting to the “*two worlds*”, which worked to ensure the continuation of cultures in the face of racism and prejudice from the dominating Western culture [86,89,96,101,103,113,116,117]. This brought the ability to achieve goals, which resulted in a more positive outlook [104,106,117].

*“…participants and workgroup members also emphasized the capacity for resiliency, adaptation, and cultural renewal evidenced by [the Indigenous peoples in the United States].”*

[117]

*“Gaining independence enhanced the well-being of many participants… Learning to adjust was essential for participants who maintained well-being.”*

[103]

##### Basic Needs

Basic needs were reported as important to the wellbeing of Indigenous peoples in the United States by 17 papers [81,86,87,90,91,94,95,96,100,101,103,105,110,111,115,117,118].

Money and employment were reported together, with other aspects of having employment not reported as being as important as earning [86,87,90,96,100,105,115,117,118]. The desire for money was to meet the needs of the family [86,87,100,103,115], such as food security and for housing [81,86,87,90,100,103,105,117]. A lack of money was seen as impacting wellbeing through the inability to purchase healthy foods and depleted mental health resulting in substance abuse [86,96,100,105,115].

*“Not having to fret about simple things like food. To be able to not lavishly but comfortably do things you’d like to do. Basically that everyone is healthy. I don’t really look at it so much as status but I mean, just being financially sort of stable… Well-being is a roof over our heads, clothes on our backs, food in our stomachs, and our health.”*

[87]

*“Her jobs were demanding, stressful, and took away from family time, all of which may have contributed to diminished well-being.”*

[103]

Services, both health and social, were considered important to wellbeing, specifically physical and mental health [81,86,87,90,91,94,95,96,100,101,110,111,117,118]. Services needed to be attuned to the individuals needs and treat them with respect, with some preferring Indigenous service providers [81,94,95,96,101,110,111,117]. Access to services was also reported as important, via location, affordability, transportation and timing [81,86,87,91,100,117,118].

*“For most of the participants, it was best if the care provider was Hawaiian or knew Hawaiian ways and respected Hawaiian culture.”*

[81]

## 4. Discussion

Our analysis of the literature identified seven themes of wellbeing of Indigenous peoples in Canada, five themes in Aotearoa (New Zealand) and seven themes in the United States (see Table 2). While we found that concepts of health and wellbeing were commonly conceived by Indigenous peoples as holistic and collectivist, wellbeing was not experienced uniformly across all Indigenous populations. These differences reflect the diverse social, political, cultural, environmental and economic contexts of Indigenous peoples’ lives [3]. Notwithstanding the marked variations in wellbeing across the populations, some shared elements emerged. Similarities, for example, in *identity*, *connection*, *balance* and *self-determination*, were found across countries.

In this review, *identity* was strongly linked to Indigenous peoples’ *connections* with family, Tribe/community, culture, the land, genealogy, and spirituality. It has been argued that these interconnected aspects of identify are integral to Indigenous peoples’ wellbeing and disruption to any of these aspects can be detrimental to Indigenous people both individually and collectively [122]. In this review, identity was commonly seen as being built and strengthened through practicing cultural ways, speaking or learning one’s Indigenous languages, and through reciprocal connections with the land and sea. Such practices have been referred to as “*acts of renewal*” [122] and, along with cultural knowledge (and Elders, as key knowledge holders), were commonly viewed by participants in included studies as major facilitators of a strong and proud identity and critical for the wellbeing of families and Tribes/communities. In all three countries, the reciprocal relationship between Indigenous peoples and the land, both in terms of the environment broadly and specifically, and their ancestral homelands, was emphasised. Indigenous people saw themselves as caretakers of the land and, in turn, saw the land as a source of physical and spiritual wellbeing, a finding also reported in the Australian review [11].

Similar to the findings of the Australian review [11], we found that *balance* of one’s mental, physical, emotional and spiritual aspects of life was seen as fundamental to Indigenous peoples’ wellbeing. Indeed, while we have separated these and other elements of wellbeing into somewhat artificial themes, it must be noted that they are interconnected and need to be considered in conjunction with each other to understand wellbeing for Indigenous peoples. In some of the included papers from Canada and the United States, Indigenous peoples’ wellbeing was conceptualised within the Medicine Wheel and its teachings. The Māori peoples of Aotearoa (New Zealand) had a similar underlying philosophy of wellbeing that also includes *whakapapa* (genealogy). *Whakapapa* encompasses connections between people, places, and times, thus linking family, land, and ancestors. Indigenous people in Canada and the United States spoke about the importance of these and described family, land and ancestors as underlying other themes of wellbeing, despite not arising as a distinct theme in itself.

*Self-determination* is a central element of wellbeing for Indigenous peoples [11,122], a finding that also emerged in the current review. While self-determination theory posits that it is important for the wellbeing of all people [123,124], for Indigenous people, self-determination extends beyond the psychological to all other aspects of life, including political, social, cultural, and economical [125]. In the current review, we found that exercising self-determination resulted in increased autonomy and agency of both the individual and the collective. An important aspect of self-determination was the ability for Tribes/communities to self-govern, based on the best interests of the Tribe/community. Self-determination was also evident through resilience in the face of ongoing colonisation, and the associated intergenerational trauma, stigmatisation and racism. Such resilience was seen to contribute to the continuation of culture, a finding echoed by Corntassel (2012), who argued that through decolonisation and resurgence “*our homelands will recognise us as being Indigenous to that place*” and future generations will have the freedom to “*live more Indigenous lives*” [122].

While there were commonalities in the themes across the three countries, it is important to note where the experiences of wellbeing diverge. For example, when looking at basic needs for individuals and Tribe/community, the Indigenous peoples in Canada and the United States reported very similar needs including adequate housing, food security and the need for culturally safe services. In Aotearoa (New Zealand), Māori people did not directly report these things as being important to their wellbeing—rather, only mentioning basic needs within the context of relationships and social networks, and how these important connections facilitated these needs. This finding echo’s those of Cram and colleagues (2003) who reported Māori conceptions of health were “*traditional*”, being holistic in nature, with the health of the *whanau* (family), *hapu* (sub-tribe) and *iwi* (tribe) stated as important [126]. This focus on the higher levels of Maslow’s Hierarchy of needs by Māori may be indicative of a widespread connection to culture, as when one is displaced from their culture, it has been found it is difficult to see past the first level of survival [127]. This is reflected in our findings also where the Indigenous people in Canada and the United States had quite varied experiences with the loss of culture, from those who felt deeply connected, to those who expressed feelings of complete disconnect.

There was an undercurrent of experiences with racism, particularly systemic and institutional racism, along with discriminatory practices that impede wellbeing in some of the included articles. This was particularly evident in terms of resilience and resistance (Canada and the United States) and also in terms of impact (e.g., on identity and power balances in Aotearoa (New Zealand)). Investigating this phenomenon and its impact on wellbeing warrants examination as a primary aim of future research.

Our review highlights the importance of understanding what constitutes wellbeing for Indigenous people. This is particularly so when developing policies and programs to achieve equity across populations. Our findings suggest wellbeing for Indigenous peoples is not completely congruent with Western biomedical views on health and wellbeing, which underpin most current measures of health and wellbeing. Angell and colleagues (2016) conducted a global systematic review of quality of life measures among Indigenous populations, and reported that only 3 out of 41 studies used Indigenous-specific measures [10]. There is also a need for wellbeing measures that prioritise the perspectives and values of Indigenous peoples [10,11,12]. In doing so, these measures can be used to make informed decisions about the allocation of public health resources to address health and wellbeing inequities for Indigenous peoples globally.

An important limitation of our study is the exclusion of grey literature. This decision was made pragmatically due to the already large scope of this review. However, as community-led projects are not always published in peer reviewed journals, the current review may not have captured these studies, nor the aspects of wellbeing reported in these. Future reviews on the topic of wellbeing in Indigenous peoples should consider inclusion of grey literature from the included countries and this should occur under the guidance of an Indigenous person from the respective country. Additionally, we note that we have included studies in our review that were led by Indigenous and non-Indigenous investigators. We recognise that this may have resulted in a somewhat different understanding of what wellbeing means to Indigenous people compared to if the review had focused exclusively on Indigenous-led studies. However, our review was led by an Indigenous Australian researcher and included guidance from senior Indigenous researchers from Australia (GG), Canada (AK), Aotearoa (New Zealand) (EW) and the United States (MC), particularly around the interpretation of findings related to their respective countries. The methods used to identify the themes described in this paper would have benefitted from having input and guidance from Indigenous peoples from the included countries from the outset. However, due to the COVID-19 pandemic and the timing of the data analysis, this was not feasible. We recommend that future reviews on Indigenous health including wellbeing be led by Indigenous persons from the respective countries, from the design through to interpretation and dissemination of findings.

## 5. Conclusions

Our review identified several themes of wellbeing important to Indigenous peoples in Canada, Aotearoa (New Zealand) and the United States. While there were some commonalities between countries, our findings reinforce the understanding that the experiences, priorities, and needs of Indigenous peoples in different places are unique and specific to local contexts. We reiterate previous findings that the conceptualisation of wellbeing for Indigenous peoples goes beyond that typically measured in quality of life measures [11]. Wellbeing measures that can accurately and reliably assess indicators of wellbeing that are important to Indigenous peoples are critically needed to inform health policy, practice and research. Future work to develop such measures needs to be led by Indigenous peoples from the outset.

## Figures and Tables

**Table 1:** Search terms.

Indigenous Population Terms	Quality of Life and Wellbeing Terms	Limiters
“American Indian *” OR “First Nation *” OR “First people *” OR Indigenous OR Inuit * OR Māori * OR Maori * OR “Native American *” OR ((Canadian OR Canada) AND Aborigin *) OR “native Canadian” OR “Indigenous population *” OR Metis OR Métis OR “Alaska * Native” OR “Native Alaska *” OR “Native Hawaiian *” OR tribal (TI/AB) ^1^	wellbeing OR well-being OR SEWB OR “quality of life” OR HR-QOL OR HRQOL OR QOL OR wellness (TI/AB) ^1^	English, Human, Peer-reviewed, research paper, inception to year 2020

^1^ TI/AB = title and abstract. * truncation symbol.

**Table 2:** Summary of themes and sub-themes of wellbeing by country.

Country	Themes	Sub-Themes	References
Canada (*n* = 43)			
	Holism/Wholism (*n* = 15)		[16,19,20,21,30,32,35,37,42,45,49,51,52,56,58]
	Culture (*n* = 33)	Identity Language	[16,19,20,21,22,25,26,27,28,29,30,31,32,33,35,36,37,38,39,40,42,43,45,47,48,49,51,52,53,54,55,56,58]
	Community and Family (*n* = 31)		[17,18,19,20,22,23,24,25,27,28,29,30,31,35,36,37,39,41,42,43,45,46,47,48,49,50,51,53,54,55,57]
	Land and Sea (*n* = 27)		[16,24,25,26,27,28,31,32,35,36,37,39,40,42,43,47]
	Resilience (*n* = 23)		[16,19,22,23,24,28,29,33,35,36,38,41,42,45,48,49,50,53,54,55,57]
	Spirituality and Cultural Medicine (*n* = 18)		[17,18,19,20,21,22,30,32,33,34,36,42,45,46,53,54,56,58]
	Physical, Mental and Emotional Wellbeing (*n* = 26)		[17,19,20,21,22,23,26,27,28,29,30,31,32,33,36,38,42,44,46,47,49,52,53,54,56,58]
Aotearoa (New Zealand; *n* = 16)			
	Māoritanga—identity (*n* = 9)		[64,66,69,70,71,72,74,75,77]
	Tikanga—Māori customs (*n* = 12)	Pūrakau (ancient story or legend) and whakatauki (proverb) Iwi (tribal) control, self-determination and autonomy Manaakitanga—to extend aroha (love/compassion) and mana (respect/power) to others Colonisation and Māori–Pākehā (European) relations	[62,63,64,65,67,69,70,71,72,73,74,75]
	Kotahitanga—togetherness and connection (*n* = 14)	Kaitiakitanga—guardianship of the land Whanaungatanga—relationships, kinships and sense of family connection	[62,63,64,65,66,69,70,71,72,73,74,75,76]
	Whakapapa—importance of genealogies (*n* = 15)	Whānau—Family Turangawaewae—sense of place	[62,63,64,65,66,67,69,70,71,72,73,74,75,76,77]
	Wairuatanga—spirituality (*n* = 8)	Rongoa—Māori medicine Karakia (prayer) waiata (songs), and tohu (signs)	[63,66,68,69,72,75,76,77]
United States (*n* = 41)			
	Holism (*n* = 10)		[87,94,95,99,101,107,108,110,112,117]
	Culture (*n* = 30)	Cultural preservation and cultural pride Language Physical activity	[79,80,81,83,85,86,88,90,91,92,93,94,95,96,97,98,99,101,103,105,106,107,111,113,114,115,116,117,118,119]
	Spirituality and Cultural Medicine (*n* = 28)	Higher power or Energy Cultural medicine	[80,81,82,85,86,87,89,90,91,92,93,94,95,97,99,100,101,104,106,107,108,110,111,112,113,117,118,119]
	Tribe/Community and Family (*n* = 29)		[79,80,81,83,84,86,87,88,89,90,95,96,97,98,99,100,101,103,104,105,106,107,112,113,114,116,117,118,119]
	Land, Sea and Subsistence-based living (*n* = 28)	Connection Sacred provider	[79,80,81,84,85,86,87,91,93,94,96,97,99,100,101,102,104,105,106,107,109,112,113,114,116,117,118,119]
	Resilience (*n* = 21)		[79,80,82,86,89,90,91,95,96,97,98,99,101,103,104,106,107,110,113,116,117]
	Basic Needs (*n* = 17)		[81,86,87,90,91,94,95,96,100,101,103,105,110,111,115,117,118]

## Data Availability

The data presented in this study are available online at the various journals all listed in the references.

## Supplementary Materials

The following are available online at https://www.mdpi.com/article/10.3390/ijerph18115832/s1, Table S1: Included papers characteristics.

## Appendix A

**Table A1:** Hierarchy table used to exclude articles and improve consistency of screening.

Exclusion Reason	If …
foreign language	Not English
population not relevant	No Indigenous adults (18+) in Canada, Aotearoa (New Zealand) and the United States included in paper
wrong publication type	Grey literature, poster abstracts, newspaper articles, case reports and dissertations, opinion piece, position statement
disease specific focus	If the study cohort, or the paper itself, has a focus on one specific disease, exclude
Systems focus	If the study is looking too narrowly at a specific system—for example a health service—then too narrow for our review
nil wellbeing focus	Doesn’t report factors of wellbeing or quality of life
review	Systematic or other reviews relevant—for checking ref lists
wrong study design	No mention of qualitative results or new empirical data in abstract, and non-relevant reviews
