# Supplementary material for: Wellbeing of Indigenous Peoples in Canada, Aotearoa (New Zealand) and the United States: A Systematic Review
Source: Int J Environ Res Public Health. 2021 May 28;18(11):5832. doi: 10.3390/ijerph18115832 (PMC8198891; doi:10.3390/ijerph18115832)
Supplement: Supplementary file 1 [file ijerph-18-05832-s001.zip › ijerph-1203896-supplementary.pdf]

Supplementary Table S1: Included papers characteristics

**Authors (year)**	**Region**	**Study Setting**	**Indigenous groups**	**Participant details**	**Was wellbeing part of main aim (YES), or component of broader research question (BROAD)?**
**Canada**					
Adelson (1998)[16]	Northern Quebec	unclear	Whapmagoostui Cree	Over 20% of the adult women and men of Whapmagoostui. Unclear if all Indigenous.	YES
Alaazi et. al. (2015)[17]	Winnipeg	unclear	Inuit, First Nations (Ojibwe & Cree, Ojibwe, Cree, Saulteaux, Dene), Métis	14 Indigenous and 6 key informant participants (age range 30-50 years). Unclear if key informants are Indigenous.	BROAD
Auger et. al. (2016)[18]	Vancouver	community/social	First Nations, Métis, Other	35 - mix of ages, genders, Aboriginal ethnicity, and roles in the community. All Indigenous.	YES
Auger (2019)[19]	British Columbia	community/social	Métis	23 women and 10 men (age range 19–84 years; mean=46). All Indigenous	YES
Bartlett (2004)[20]	Manitoba	community/social	Métis	17 Metis women - Elders in 1 focus group (mean age 59 years) and 1st (mean age 36 years) and 2nd generation (mean age 31 years) urban-dwelling adults in another. All Indigenous.	YES
Bartlett (2005)[21]	Manitoba	community/social	Métis	17 Metis women - Elders in 1 focus group (mean age 59 years) and 1st (mean age 36 years) and 2nd generation (mean age 31 years) urban-dwelling adults in another. All Indigenous.	YES
Baskin & Davey (2015)[22]	Toronto	community/social	Aboriginal	10 seniors and 2 Elders - all women (age range 60-75 years). All Indigenous.	YES

Brooks-Cleator et. al. (2019)[23]	Ottawa	community/social	First Nations, Inuit	32 Indigenous older adults (23 Inuit; 8 males, 15 women) 9 First Nations (3 males, 6 women) (age range 55-79 years). All Indigenous.	BROAD
Castleden et. al. (2016)[24]	Pictou Landing First Nation	community/social	Mi'kmaw	10 Elders - 8 women. All Indigenous.	YES
Condon et. al. (1995)[25]	Holman	community/social	Copper Inuit	20 young male household heads (age range 23-35 years). All Indigenous.	BROAD
Cunsolo Willox et. al. (2012)[26]	Rigolet, Nunatsiavut	community/social	Rigolet Inuit	43 women, 29 males (age range 9-85 years). All Indigenous.	YES
Fillion et. al. (2014)[27]	Inuvialuit Settlement Region	community/social	Inuit	23 participants from Inuit organizations and community representatives, university-based researchers from the Inuit Health Survey and Northwest Territories governmental organisations. Some Indigenous and some non-Indigenous.	BROAD
Fraser et. al. (2018)[28]	Nunavik, Quebec	community/social	Inuit - Nunavimmiut	2 elders (women), 11 mothers, and 1 father (age range 20-65 years). All Indigenous.	YES
Fraser et. al. (2019)[29]	Nunavik, Quebec	community/social	Inuit - Nunavimmiut	2 elders (women), 11 mothers, and 1 father (age range 20-65 years). All Indigenous.	YES
Gone (2011)[58]	Northern Canada	hospital/health centre/place of healing	First Nations	19 staff and clients - The current and former executive directors of the Lodge, the counseling program coordinator, and a member of the counseling program's oversight committee (3 women, all middle aged - unclear if Indigenous). 4 current and former counselors (2 male, 2 woman, aged early 40s to late 60s - all Indigenous). 11 clients who had already concluded their treatment (7 men, 4 female, age ranged from 20-60s - all Indigenous).	BROAD
Graham & Martin (2016)[30]	Thunderchild First Nation, Saskatchewan	multiple settings	néhiyawak (Plains Cree people)	15 participants (age range 18-71 years); 7 male and 8 women. 3 Elders. All Indigenous.	YES
Harper et. al. (2015)[31]	Nunatsiavut, Labrador	community/social	Inuit	11 government employees (2 male, 9 women, age range 21-50+ years). Photovoice with 11 community members (5 women, 6 male). Community members Indigenous - unsure about government employees.	BROAD

Hatala et. al. (2016)[33]	Saskatchewan	community/social	Cree	4 Canadian Cree Elders - 2 males, 2 women (age range 53-83 years). All Indigenous.	YES
Hatala et. al. (2019)[32]	Saskatoon, Saskatchewan	community/social	Plains Cree and Metis	28 youth - 12 male, 16 women (age range 15-25 years), 21 nêhiyaw (Plains Cree) and 7 Métis. All Indigenous.	YES
Keightley et. al. (2011)[34]	Kenora, Ontario	community/social	Ojibway	10 participants - range of Elders, traditional healers and mental health case workers. 7 male and 3 women (age range 30-70 years) - 8 Indigenous, 1 English and 1 unknown.	BROAD
Kral et. al. (2011)[35]	Nunavut	community/social	Inuit	50 Inuit (age range 14–94 years), 25 women and males. All Indigenous.	YES
Kyoon-Achan et. al. (2018)[36]	Manitoba	community/social	Eight First Nation communities representing four First Nation languages; (Ininiwak-Cree, Dakota, Dene and Ojibway or Anishnaabe)	61 participants - 54 interviews and 1 focus group. 7 participants in focus group, inluding 3 workers who's Indigenous status unknown - remainder Indigenous.	YES
Lemelin et. al. (2010)[37]	Hudson Bay lowlands, Ontario	community/social	Cree	12 Cree harvesters (8 men and 4 women), and 10 Elders (6 men and 4 women). All Indigenous	YES
Matheson et. al. (2020)[38]	All	multiple settings	First Nations, Metis	134 participants – Group one 78 (57 women, 21 males; age mean=41.62 years; SD=11.15). Group two 56 (47 women, 9 males; age mean=36.62; SD=11.91). All Indigenous.	YES
Mikraszewicz & Richmond (2019)[39]	Pic River, Ontario	community/social	Biigtigong Nishnaabeg	4 adults and 5 youth. All Indigenous	YES
Moore (2019)[40]	Nunatsiavut	school/university/workplace	Inuit	5 Inuit women (age range 28-55 years). All Indigenous	BROAD
Motz & Currie (2019)[41]	Lethbridge, Alberta	school/university/workplace	First Nations, Metis, Inuit	142 participants - 63.1% of the sample identified as First Nations, 23.2% as Indigenous generally (without a specific affiliation), and 13.5% as Métis. All Indigenous.	BROAD

O’Neil et. al. (2016)[42]	British Columbia	multiple settings	First Nations	14 from First Nations Health Council, 7 from First Nations Health Directors Association and 13 from provincial health system. Policy roundtable with 60 participants. Some Indigenous and some non-Indigenous.	BROAD
Pace & Gabel (2018)[43]	Newfoundland and Labrador	community/social	Southern Inuit	5 Southern Inuit older adults (age range 50–75 years, all women) and 5 youth (age range 8–24 years; 2 women and 3 male). All Indigenous.	BROAD
Parker et. al. (2019)[44]	Sioux Lookout, Ontario	community/social	First Nations	Approximately 100 participants. All Indigenous.	BROAD
Parlee et. al. (2005)[46]	Fort McPherson, Northwest Territories	community/social	Teetl’it Gwich’in	75 women. All Indigenous.	BROAD
Parlee et. al. (2007)[45]	Lutsel K'e, Northwest Territories	community/social	Dene	Home-visit interviews with each household in the community. All Indigenous.	YES
Petrasek MacDonald et. al. (2015)[47]	Nunatsiavut, Labrador	community/social	Inuit	17 youth (age range 15–25 years). All Indigenous.	YES
Richmond et. al. (2005)[48]	Alert Bay, British Columbia	community/social	Namgis First Nation	Community sample: 15 employed and 4 unemployed members, and 4 key informants (age range 25-72 years). All community members Indigenous – unsure about key informants.	YES
Schill et. al. (2019)[49]	Kelowna, British Columbia	community/social	Urban Indigenous Canadians	Preliminary sharing circle - 6 community members and 2 community stakeholders. Interviews 9 urban Indigenous adults (age 55+). Final sharing circle - 7 community Elders and 2 other. All Indigenous.	YES
Spiegel et. al. (2020)[50]	British Columbia	community/social	Tsleil-Waututh Nation	4 Elders and 4 younger members of the Tsleil-Waututh Nation. All Indigenous.	BROAD
Stewart (2008)[51]	Nil data	unclear	First Nations or Metis	5 First Nations or Metis individuals. All Indigenous.	BROAD
Tam et. al. (2013)[52]	James Bay, Ontario	community/social	Fort Albany First Nation	39 community members - 17 males, 22 women (age range 22-73 years; mean=42). All Indigenous.	YES

Thompson et. al. (2013)[53]	2 First Nations service centers: 1 metropolitan, 1 northern city	community/social	First Nations	15 First Nations grandparents - 14 women, 1 male. All Indigenous.	YES
Tobias & Richmond (2014)[54]	North Shore of Lake Superior	community/social	Anishinaabe, The Batchewana First Nation of Ojibways, and The Ojibways of the Pic River First Nation	46 Elders. All Indigenous.	BROAD
Waddell et. al. (2017)[55]	Cape Dorset, Nunavut	community/social	Inuit	6 male, 4 women. All Indigenous.	YES
Wilson (2003)[56]	Northern Ontario	community/social	Anishinabek (Ojibway and Odawa)	14 community members (9 women, 5 men). All Indigenous.	BROAD
Zurba & Bullock (2019)[57]	Nationwide - mainly British Columbia & Ontario	unclear	First Nations, Metis, Inuit, Settler, Other.	13 male, 2 women, 3 no response (age range 30-60+ years). All Indigenous.	YES
**Aotearoa (New Zealand)**					
Beavis et. al. (2019)[62]	Wellington and Northland	community/social	Māori	4 Māori households (18 individuals) - 7 adults - 4 women, 3 male (age range 32-65 years) 11 children present (age range 2-43 years). All Indigenous.	YES
Bell et. al. (2017)[63]	Nil data	community/social	Māori	7 male (age range 28-56 years), 8 women (age range 25-49 years). All Indigenous	YES
Butcher & Breheny (2016)[64]	Eastern Bay of Plenty	community/social	Māori	8 older Māori (66-79 years old). All Indigenous.	BROAD
Hapeta et. al. (2019)[65]	Bay of Plenty	community/social	Māori, Pasifika, Pākehā	Head coach (Indigenous), team members and staff of one Rugby team - (18 players and 6 coaches). Some Indigenous and some non-Indigenous.	YES

Hopkirk & Wilson (2014)[66]	Nil data	hospital/health centre/place of healing	Māori	2 Māori occupational therapists, 1 New Zealander occupational therapist and 2 Māori health specialists. 4 Indigenous, 1 non-Indigenous.	YES
Lawson-Te Aho et. al. (2019)[67]	Nil data	community/social	Māori	20 participants - included Māori researchers, Te Tiriti experts, Kaumātua/ esteemed elders; researchers, academics, Māori homelessness activists and experts in decolonisation and re-indigenisation. All Indigenous.	BROAD
Mark & Lyons (2010)[69]	Auckland and 2 other rural towns	community/social	Māori	6 Māori spiritual healers - 5 women and 1 male. All Indigenous.	YES
Mark & Lyons (2014)[68]	Auckland and 2 other rural towns	community/social	Māori	12 spiritual healers - 6 Māori (5 women, 1 male) and 6 non-Māori. Some Indigenous and some non-Indigenous.	BROAD
Raerino et. al. (2013)[70]	Auckland	unclear	Māori	12 women and 7 men (age range 18-75+ years). All Indigenous.	YES
Rata et. al. (2008)[71]	Taranaki & Wellington	community/social	Māori	10 Māori – 2 women, 8 male (age range 20-75 years; mean=42). All Indigenous.	BROAD
Rawson (2016)[72]	Christchurch	unclear	Māori	Four focus groups 8-12 participants each, 1 key stakeholder interview. Mix of families and elders, students, parents and staff from a Māori school. All Indigenous.	YES
Roche et. al. (2018)[73]	Various localities (Auckland, Wellington, Christchurch, Hamilton, Dunedin, plus regional)	community/social	Māori	18 Māori leaders - 8 male and 10 women. All Indigenous.	YES
Rua et. al. (2017)[74]	Hamilton, Ruatoki, Whakatone	community/social	Māori	5 Māori men (age range 36-45 years). All Indigenous.	BROAD

Spiller et. al. (2011)[75]	Nil data	unclear	Māori and Pākehā	54 interviews – 4 businesses:<br>. Trust owned by people of hapu, subtribe<br>. Joint venture Māori and Pākehā<br>. Private company owned by Māori<br>. Pākehā owners, business promoted Māori cultural tourism experiences, employed Māori guides, and was mentored by kaumatua, elders. Some Indigenous and some non-Indigenous.	YES
Willing et. al. (2019)[76]	Across NZ	unclear	Māori	4 women, 2 male (age range 25-55+ years including 2 Elders). All Indigenous.	BROAD
Wilson (2008)[77]	Nil data	community/social	Māori	38 Māori women (age range 24-61 years). All Indigenous.	YES
**United States**					
Andrade et. al. (2019)[79]	Washington State, Arizona, Georgia	multiple settings	American Indian, Alaska Natives	10 Indian Health Service Cultural advisors, 3 Tribal Advisory Committee members, 3 Centres for Disease control staff, 1 Substance Abuse and Mental Health Services Administration member and 3 American Indian facilitators. Some Indigenous and some non-Indigenous.	YES
Ayunerak et. al. (2014)[80]	Southwest Alaska	multiple settings	Yup'ik Inuit	4 author perspectives - 2 elders and 2 local prevention workers. All Indigenous.	BROAD
Browne et. al. (2014)[81]	Hawai'i	community/social	Native Hawaiians	24 Elders (21 women, 3 male; age range 60-94 years) 17 caregivers (11 women, 6 male; age range 38-77 years). All Indigenous.	YES
Buehler (1992)[82]	South Central Montana	community/social	Crow Indians	13 Crow Indians, 5 non-Indian nurses and 2 non-Indian physicians associated with the Indian Health Service. Some Indigenous and some non-Indigenous.	BROAD
Burnette (2018)[83]	Multiple rural, federally recognised Indigenous reservation communities in	community/social	Indigenous peoples of the US	29 Indigenous women (age range 22-74 years; mean=40), 20 health professionals (11 male; age range 26-76 years; mean=49). Some Indigenous and some non-Indigenous.	BROAD

	the southeastern United States.				
Burnette et. al. (2018)[84]	Southeastern United States.	community/social	Indigenous peoples of the US	436 participants - 254 interviews, 217 participated in 27 focus groups, and 163 family interviews. Participants included professionals who worked with Indigenous peoples, elders (age 55 or older), adults (ages 24–54), and youth (ages 11–23). Some Indigenous and some non-Indigenous.	YES
Carroll et. al. (2018)[85]	Oklahoma	community/social	Cherokee Nation	Cherokee Nation Medicine Keepers 11 elders (5 women; 6 men). All Indigenous	BROAD
Christiansen et. al. (2019)[86]	American Southwest and Upper Mid-west	community/social	Ojibwa, Potawatomi, Navajo	89 women - primary caregivers and tribal employees from tribal agencies, schools, and enterprises. All Indigenous.	BROAD
Danes et. al. (2016)[87]	Minnesota	unclear	American Indians from Northern Ojibwe	11 women, 4 male. All Indigenous.	BROAD
Deacon et. al. (2011)[88]	South Central Oklahoma	unclear	Chickasaw Nation	7 key informant employees at the Division of History and Culture of the Chickasaw. Various participants from a Search Conference (participatory planning meeting with stakeholders) – Some Indigenous and some non-Indigenous.	YES
Elm et. al. (2016)[89]	Nil data	unclear	American Indian/Alaska Native	11 women - age range 20s to late 50s. Some Indigenous and some non-Indigenous.	BROAD
Friesen et. al. (2015)[90]	Nil data	unclear	American Indian/Alaska Native	Grup one: 15 women, 3 men. Group two: 6 women, 9 men. 2 youth advocates and 4 young adults. Age range 17-24 years. All Indigenous.	BROAD
Goodkind et. al. (2015)[91]	Navajo Nation	multiple settings	Dine	14 youth (6 male, 8 women; age range 12-17 years), 15 parents/guardians (12 women, 3 male; age range 24-49 years), and 8 elders (all women; age range 54-90 years). Some Indigenous and some non-Indigenous.	YES
Grayshield et. al. (2015)[92]	Western, Southwestern,	community/social	Native American	11 Native American elders - 8 male, 3 women (age range 54-90 years). All Indigenous.	BROAD

	and upper Midwest USA				
Griffin-Pierce (1997)[93]	Colorado	school/university/workplace	Navajo	3 women, 1 male, University students. All Indigenous	YES
Hilgendorf et. al. (2019)[94]	Northeast Wisconsin	community/social	Menominee Nation	5 Menominee Wellness Initiative members in interviews, 12 in focus groups. Some Indigenous and some non-Indigenous.	YES
Hodge & Limb (2010)[95]	Whole country	community/social	Native American - Lakota, Navajo/Dine, Chippewa/Ojibwa, Cherokee, Other	50 Native American experts - 32 women, 18 male (age mean=49.2 years). 8 non-Native participants, others all Indigenous.	BROAD
Hulen et. al. (2019)[96]	Flagstaff, Arizona	community/social	American Indian	39 American Indian (8 male, 31 women) 22 health care providers (7 men, 15 women). Some Indigenous and some non-Indigenous.	YES
Isaacson et. al. (2018)[97]	Northern Plains	community/social	Native American	6 Native American Elders (2 male, 4 women) 8 Native American youths (7 women, 1 male). All Indigenous.	YES
Jacob et. al. (2019)[98]	Nil data	school/university/workplace	Native American - 2 from Northwest Tribes and one from a Southwest Tribe	3 women students. All Indigenous.	BROAD
Kading et. al. (2019)[99]	Nil data	unclear	American Indian and First Nations (Anishinaabe)	13 Anishinaabe (age range 19–29 years). All Indigenous.	YES
Kodish et. al. (2016)[100]	California	unclear	American Indian	12 Tribal leaders (4 male, 8 women) 24 tribal members (4 male, 20 women). All Indigenous.	BROAD
Lane (2018)[101]	Standing Rock, Dakota	community/social	Native American	3 women. All Indigenous.	BROAD
Lassetter (2011)[102]	Las Vegas	community/social	Native Hawaiian	27 participants (11 women, 16 males; age range 23-62 years, mean=40). All Indigenous.	YES
Lassetter et. al. (2012)[103]	Las Vegas	community/social	Native Hawaiian	27 participants (11 women, 16 males; age range 23-62 years, mean=40). All Indigenous.	YES

Lewis (2011)[106]	Bristol Bay, Southwest Alaska	community/social	Alaska Native - Yup'ik Eskimo, Aleut, Athabascan	26 Alaska Native Elders (age range 61-93 years). All Indigenous.	BROAD
Lewis (2013)[104]	Bristol Bay, Southwest Alaska	community/social	Alaska Native - Yup'ik Eskimo, Aleut, Athabascan	26 Alaska Native Elders (age range 61-93 years). All Indigenous.	BROAD
Lewis (2014)[105]	Bristol Bay, Southwest Alaska	community/social	Alaska Native - Yup'ik Eskimo, Aleut, Athabascan	25 Alaska Native Elders (age range 61-93 years), 14 women, 11 male. All Indigenous.	YES
Lewton & Bydone (2000)[107]	Nil data	unclear	Navajo	Number not reported - Navajo healers and their patients affiliated with Traditional Navajo religion, the Native American Church, and Pentecostal Christianity. Some Indigenous and some non-Indigenous.	BROAD
Look et. al. (2014)[108]	5 different Hawaiian Islands	community/social	Native Hawaiian	6 prominent kumu hula from 5 different Hawaiian Islands (1 male, 5 women). All Indigenous.	YES
Mitchell (2018)[109]	Midwestern United States	community/social	American Indian	9 female, 2 male (age range 25-74 years). All Indigenous.	BROAD
Moghaddam et. al. (2015)[110]	Midwestern United States	multiple settings	American Indians and Alaska Natives	27 Community members (18 women, 9 male; age range 12–82 years) 11 service providers (5 male, 6 women; age range 26–70 years). 30 Indigenous and 8 non-Indigenous.	BROAD
Moorehead et. al. (2015)[111]	Michigan	unclear	Native American	18 traditional healers, clinically trained service providers, and cross-cultural mental health researchers. Some Indigenous and some non-Indigenous.	BROAD
Odom et. al. (2019)[112]	Honolulu, Hawaii	community/social	Native Hawaiian	25 women, 13 men. Some Indigenous and some non-Indigenous.	BROAD
Oneha (2001)[113]	Wai'anae, Hawai'i	community/social	Native Hawaiian	13 men and women (age range 36-80 years). All Indigenous.	BROAD

Shea et. al. (2019)[114]	Oklahoma	multiple settings	Miami Tribe	32 Myaamia college students (59% female, 41% male, age mean=20), 6 Myaamia tribal seniors (all female, mean age=22) and 800 community members (observation data only). Unclear how many community members were Indigenous.	BROAD
Skewes & Blume (2019)[115]	Montana	community/social	American Indians and Alaska Natives	13 men, 12 women (age range 28–79 years). All Indigenous.	BROAD
Trout et. al. (2018)[116]	Arctic Alaska	unclear	Inupiaq Alaskan Native	11 Inupiaq youth (age range 14-22 years), 10 adults, 6 University students (age range 19-25 years). Some Indigenous and some non-Indigenous.	BROAD
West et. al. (2012)[117]	Chicago	community/social	American Indian	107 American Indian youth and families (66% women, 41% under age of 25). All Indigenous.	BROAD
Williamson et. al. (2019)[118]	Nil data	community/social	Native American	4 IDD males (age range 19-24 years) 4 caregivers (3 fwomen, 1 male; age range 38-63 years). All IDD participants Indigenous, unclear if carers were.	YES
Wolsko et. al. (2006)[119]	Southwestern Alaska	community/social	Yup'ik Eskimos	64 Yup'ik adults (age rang ~18-50+ years). All Indigenous.	YES
